# The non-classical nuclear import carrier Transportin 1 modulates circadian rhythms through its effect on PER1 nuclear localization

**DOI:** 10.1371/journal.pgen.1007189

**Published:** 2018-01-29

**Authors:** Sandra Korge, Bert Maier, Franziska Brüning, Lea Ehrhardt, Thomas Korte, Matthias Mann, Andreas Herrmann, Maria S. Robles, Achim Kramer

**Affiliations:** 1 Laboratory of Chronobiology, Charité—Universitätsmedizin Berlin, Berlin, Germany; 2 Department of Proteomics and Signal Transduction, Max Planck Institute of Biochemistry, Martinsried, Germany; 3 Institute of Medical Psychology, LMU Munich, Munich, Germany; 4 Molecular Biophysics, Department of Biology, Humboldt Universität zu Berlin, Berlin, Germany; Center for Integrative Genomics, SWITZERLAND

## Abstract

Circadian clocks are molecular timekeeping mechanisms that allow organisms to anticipate daily changes in their environment. The fundamental cellular basis of these clocks is delayed negative feedback gene regulation with PERIOD and CRYPTOCHROME containing protein complexes as main inhibitory elements. For a correct circadian period, it is essential that such clock protein complexes accumulate in the nucleus in a precisely timed manner, a mechanism that is poorly understood. We performed a systematic RNAi-mediated screen in human cells and identified 15 genes associated with the nucleo-cytoplasmic translocation machinery, whose expression is important for circadian clock dynamics. Among them was Transportin 1 (TNPO1), a non-classical nuclear import carrier, whose knockdown and knockout led to short circadian periods. TNPO1 was found in endogenous clock protein complexes and particularly binds to PER1 regulating its (but not PER2’s) nuclear localization. While PER1 is also transported to the nucleus by the classical, Importin β-mediated pathway, TNPO1 depletion slowed down PER1 nuclear import rate as revealed by fluorescence recovery after photobleaching (FRAP) experiments. In addition, we found that TNPO1-mediated nuclear import may constitute a novel input pathway of how cellular redox state signals to the clock, since redox stress increases binding of TNPO1 to PER1 and decreases its nuclear localization. Together, our RNAi screen knocking down import carriers (but also export carriers) results in short and long circadian periods indicating that the regulatory pathways that control the timing of clock protein subcellular localization are far more complex than previously assumed. TNPO1 is one of the novel players essential for normal circadian periods and potentially for redox regulation of the clock.

## Author summary

Circadian clocks are endogenous timekeeping mechanisms allowing organisms to anticipate daily changes in their environment. In mammals, the fundamental mechanism of these clocks is a delayed negative feedback loop, in which timely auto-repression of clock components is essential. This repression occurs at a transcriptional level and requires clock proteins to enter the nucleus in a precisely timed manner, a regulation that is little understood. We performed a systematic genetic screen for factors modulating subcellular localization in oscillating human cells and identified Transportin 1 (TNPO1) as a non-classical carrier protein required for a normal circadian period. The primary target of TNPO1 within the circadian clockwork is PERIOD1, whose nuclear shuttling is modulated by TNPO1. In addition, TNPO1-mediated nuclear import may constitute a novel input pathway of how cellular redox state signals to the clock, since redox stress increases binding of TNPO1 to PER1 and decreases its nuclear localization.

## Introduction

Circadian clocks are endogenous oscillators that have evolved in almost all eukaryotes to anticipate daily rhythms in their environment. In mammals, circadian rhythm generation is a cell-autonomous process with transcriptional-translational feedback loops as fundamental mechanism [1]. A key step is the rhythmic inhibition of the CLOCK-BMAL1 transactivation activity by a negative feedback complex that contains PER and CRY proteins [2], which thereby inhibit their own expression. Circadian oscillations only occur because this negative feedback is delayed, *i*.*e*. after complex maturation, modification and nuclear translocation of PER and CRY proteins. While it is widely accepted that the regulation of subcellular localization of negative feedback components is a critical step for the generation of a normal near-24-hour period, our understanding of the mechanisms of nucleo-cytoplasmic translocation of mammalian clock proteins is limited.

Several studies highlighted the importance of posttranslational modifications (PTMs) of clock proteins for the regulation of subcellular localization. For example, oscillations in nuclear abundance of CLOCK-BMAL1 are largely dominated by rhythmicity of BMAL1 protein and its PTMs, since overall CLOCK levels are barely rhythmic and the absolute abundance of BMAL1 protein seems to be much lower than of CLOCK [3]. Thus, BMAL1 levels are likely rate limiting for CLOCK-BMAL1 nuclear abundance. Indeed, BMAL1 is required for nuclear localization of CLOCK [4], and nucleo-cytoplasmatic shuttling of BMAL1 seems to play an essential role for it [5]. A critical signal in this context is the phosphorylation of BMAL1 at Ser90 mediated by CK2α [6], which promotes CLOCK-BMAL1 heterodimerization and—probably thereby—nuclear accumulation of both proteins.

Circadian inhibition of CLOCK-BMAL1 transcriptional activity is fundamentally determined by the precisely timed activity of the PER/CRY complex. This nuclear complex has been found to be bigger than 1 MDa [7, 2] and consists–in addition of PER and CRY proteins–of several additional proteins that are likely recruited to the CLOCK/BMAL1 heterodimer to contribute to transcriptional shutdown. Thus, the timing of nuclear localization and activity of this complex as well as its inactivation is of critical importance for circadian period. PTMs of PER and CRY proteins play dominant roles in this context. PER and CRY proteins are heavily phosphorylated in a circadian manner [3] regulating their subcellular localization as well as stability, which ultimately impacts in the nuclear activity of the PER/CRY complex [8–13]. As it is the case for CLOCK and BMAL1, also PER and CRY proteins support each other’s nuclear localization [3, 14, 15], thus anything affecting PER or CRY protein stability will likely also influence the activity of the inhibitory complex as a whole. PER proteins are the rate limiting component of the PER/CRY complex [3, 16] and have been suggested to be primarily responsible for the timely nuclear accumulation of the dominant CLOCK/BMAL1 repressors–the CRY proteins [17–19]. Thus, PER stability as well as nuclear accumulation dynamics is of utmost importance for circadian rhythm generation.

These studies demonstrate the critical role of timely nuclear localization of clock proteins for circadian rhythm generation. The precise molecular mechanism, by which nuclear localization of clock proteins occurs, however, is very little understood. Because of their size and the fact that they form various complexes already in the cytosol [2], clock proteins cannot passively diffuse into the nucleus but have to be actively transported through the nuclear pore *via* nuclear import carriers. The predominant, “classical” nuclear import pathway involving Importin α and β, which recognize so-called classical nuclear localization signals (cNLS), seems to play an important role in this context. Within the last two decades several functional cNLS were identified in clock proteins [20–23]. Indeed, misregulation of Importin α2 (KPNA2) impairs clock development and alters the localization of PER proteins [24]. Also RNAi-mediated downregulation of Importin β (KNPB1) affects circadian dynamics probably by decreasing nuclear localization of PER and CRY proteins [25].

While these studies focused on specific components of the classical nuclear import pathway, the fact that ~60 different proteins comprise the nucleo-cytoplasmic translocation machinery, suggests a much more complex regulation. Here, we present a systematic approach to test the impact of 62 genes involved in nuclear-cytosolic translocation for circadian rhythm generation. Using RNAi-mediated knockdown and live cell bioluminescence recording of circadian rhythms in reporter cells, we identify fifteen of those genes whose expression is essential for normal circadian dynamics. Among them was Transportin 1 (TNPO1), a non-classical nuclear import carrier, whose knockdown or knockout led to short circadian periods. One cargo of TNPO1 is PER1; it interacts with TNPO1, and its (but not PER2’s) nuclear accumulation as well as nuclear import kinetics is reduced upon *Tnpo1* knockdown. Interestingly, oxidative stress conditions increased binding between TNPO1 and PER1 and impaired nuclear PER1 import suggesting that TNPO1-mediated PER1 nuclear localization is a redox-sensitive input into the circadian clock.

## Results

### The non-classical nuclear import carrier TNPO1 regulates circadian rhythms

**Fig 1:**
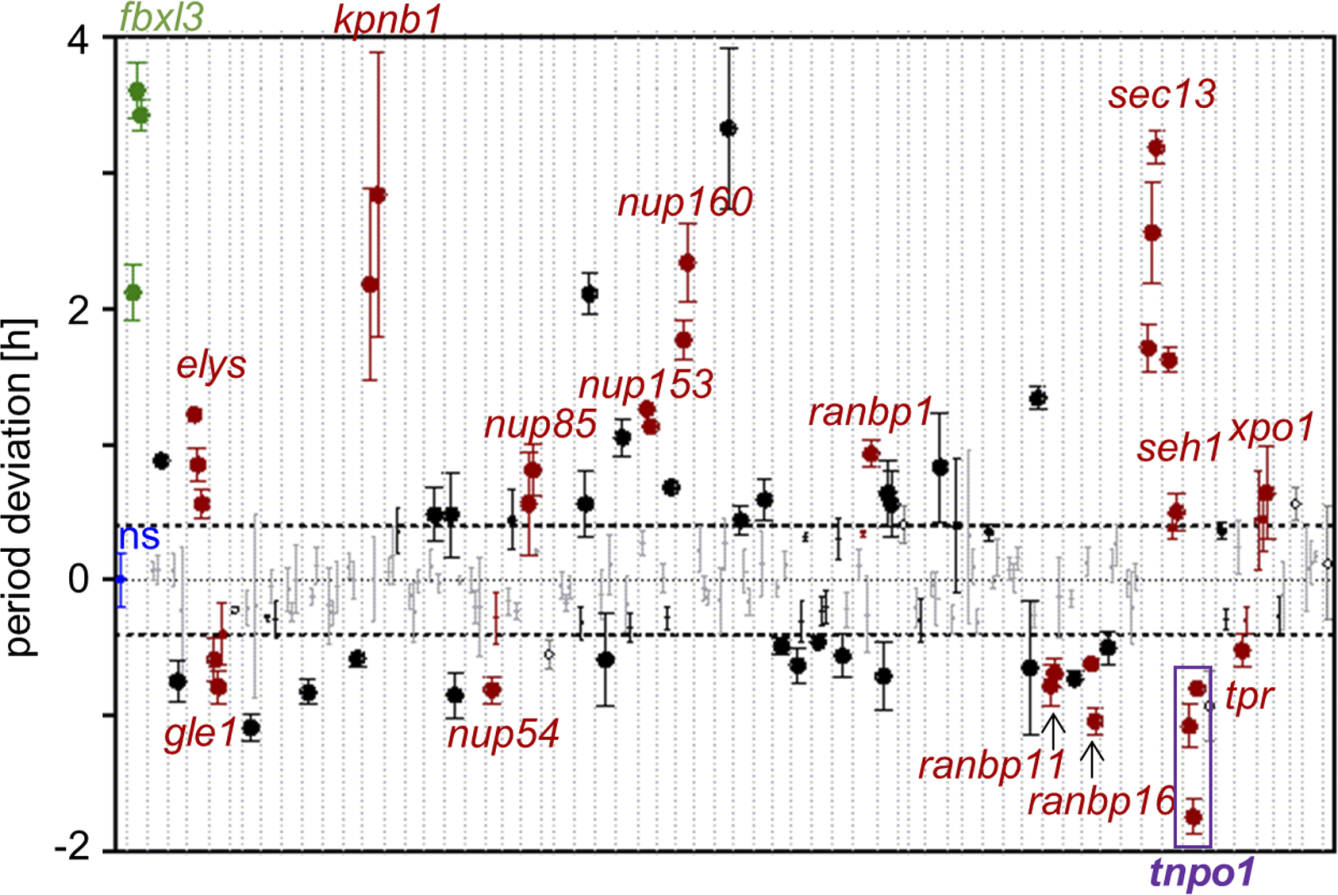
An RNAi screen identifies genes associated with the nucleo-cytoplasmic translocation machinery important for circadian dynamics. U-2 OS *Bmal1-*luciferase reporter cells were transduced with RNAi constructs targeting 62 genes associated with nucleo-cytoplasmic translocation and bioluminescence rhythms were recorded for several days. Significant period alteration compared to non-silencing controls (ns, blue) was determined by one-way ANOVA of all constructs targeting one gene; a Dunnetts posttest assessed the effects of individual constructs. Multiple testing was corrected for by a Bonferroni-Holm correction. Horizontal dotted lines indicate two standard deviations of the ns control period. Targeting *Fbxl3* was used as positive control (green). Genes for which at least two shRNA constructs led to a significant period alteration in the same direction are labeled (red dots); non-significant values are depicted in grey; dot size indicates the significance of period deviation: smallest: 0.05 > q > 0.005, medium: 0.005 > q > 0.001, largest: q < 0.001). When only one RNAi construct was available for gene knockdown, period deviation is depicted as empty circle (error bars = SD, n = 3).

**Fig 2:**
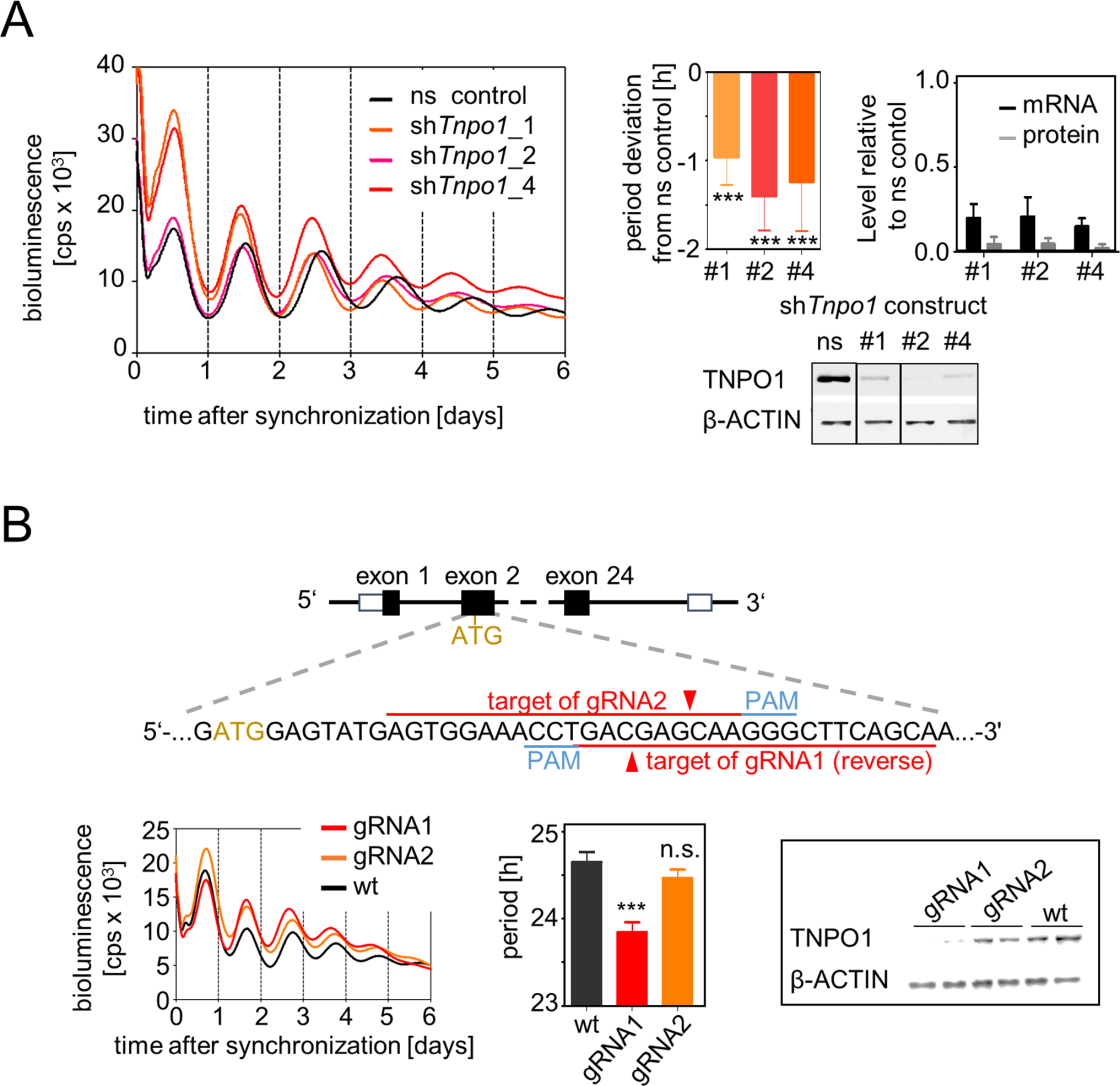
Knockdown and knockout of TNPO1 shortens the circadian period. (**A)** Left: Representative time series for the effect of three different sh*Tnpo1* constructs compared to a non-silencing (ns) control shRNA. Right: Mean period deviation and relative *Tnpo1* mRNA and protein levels. Bottom: representative western blot of TNPO1 protein levels in ns control and *Tnpo1* depleted cells; lanes are from the same blot with identical exposure time. Error bars = SD, n_period deviation_ = 7 to 14 individual recordings, n_mRNA and protein_ = 4–10 individual cell lysates; one-sample t-test to test whether period deviations are different from zero, *** p < 0.001. (**B)** Top: Schematic illustration of the CRISPR/Cas9-mediated genome editing of the *Tnpo1* gene. Bottom left: Representative time series of U-2 OS reporter cells transduced with Cas9 expression vector and indicated guide RNA (gRNA). Middle: Mean periods of independent bioluminescence recordings (error bars = SD, n = 3, one-way ANOVA with Bonferroni posthoc test comparing sgRNAs to wild-type: *** p < 0.001, n.s. = non- significant. Right: Representative western blot of residual TNPO1 protein levels in genome-edited cells.

To identify nucleo-cytoplasmic translocation associated genes essential for circadian dynamics we RNAi-knocked down the expression of 62 genes of the nucleo-cytoplasmic translocation machinery. These included 29 nuclear pore complex components as well as 27 nuclear import and export carriers. Human osteosarcoma (U-2 OS) reporter cells—an established and robust cellular clock model—expressing luciferase from a *Bmal1* promoter fragment were transduced with up to three shRNA constructs per target gene, and circadian luciferase activity rhythms were monitored over a period of one week. Fifteen genes turned out to be essential for normal ~24 hour period length. The depletion of nine of them led to period lengthening and six of them to period shortening (**Fig 1 and S1 Table**). Interestingly, while the genes whose knockdown led to longer periods included the classical import carrier *Kpnb1* (also known as Importin β), depletion of the alternative nuclear import carrier *Tnpo1* (also known as Karyopherin β2) led to an opposite period phenotype shortening the circadian period by 1–1.5 hours (**Fig 2A**). In addition, knockdown of *Tnpo1* altered clock gene expression resulting in early phases of circadian rhythms and–in particular for the *Period* genes–also in reduced overall expression levels (**S1 Fig**). Together, these data suggest the existence of a yet unknown nuclear import pathway important for circadian oscillations.

To validate these results, we created a total *Tnpo1* knockout cell line using CRISPR/Cas9 genome editing technology. We lentivirally transduced U-2 OS reporter cells with CRISPR/Cas9 vectors harboring different guide RNAs (gRNA1 and gRNA2), selected for positively transduced cells and tested circadian dynamics. One of the two tested guide RNAs (gRNA1) caused a significant period shortening of the cell population by ~1 hour (gRNA1; p < 0.001) as well as an efficient depletion of TNPO1 protein, while gRNA2 was ineffective with regard to both period shortening and protein depletion (**Fig 2B**). To test, whether we obtained a total knockout of the *Tnpo1* gene in the gRNA1 targeted cells, we performed limited dilution and sequenced the single cell clones to detect insertions or deletions (indels) causing shifts of the open reading frame. From the identified six single cell clones with indels on both *Tnpo1* alleles, only one had indels causing frame shifts that lead to premature STOP codons in the open reading frame of *Tnpo1*. As expected from the cell population, this total knockout clone also displayed ~1–1.5 hour shorter circadian rhythms as well as no detectable TNPO1 protein expression (**S2 Fig**).

### PER and CRY proteins contain functional TNPO1 recognition motifs

In contrast to classical nuclear localization signals, most TNPO1 cargoes contain non-classical motifs, so called M9 NLSs. While M9 NLSs are less well defined compared to classical NLSs, most M9 NLSs contain a PY motif (23 out of 24 known TNPO1 cargos; [26]). In addition, it has been shown that TNPO1 interaction to e.g. FOXO4 is independent of M9 NLSs and rather mediated by covalent intermolecular disulfide bridges [27]. If the period shortening upon *Tnpo1* depletion is a direct effect on the core circadian oscillator, canonical clock proteins might be direct targets of TNPO1 and thus might contain recognition motifs in their primary structure. To identify potential TNPO1-binding sites in circadian clock proteins, we searched for putative M9 NLSs in the protein sequences of BMAL1, CLOCK, CRY1, CRY2, PER1 and PER2. In addition to classical NLSs, five of the investigated clock proteins contain at least one evolutionary conserved PY motif (CRY1, CRY2, PER1, PER2 and BMAL1; **S2 Table**). Due to the lack of structural knowledge, for many of these motifs it is unknown, whether they are surface-exposed in the native protein. Nevertheless, we tested, whether these sequences (40–50 amino acids) are in principle able to promote nuclear localization in a TNPO1-dependent manner. To this end, we expressed them as fusion proteins with cyan or yellow fluorescent protein (CFP or YFP) and analyzed the subcellular localization of these fusion proteins in U-2 OS and HEK293 cells either with or without endogenous TNPO1. While six of the ten investigated clock protein-derived PY-peptides could not drive CFP in the nucleus, four PER and CRY peptides promoted CFP nuclear localization similar to a positive control peptide derived from the known TNPO1 cargo hnRNP A1 [28]. These effects were at least in part TNPO1-dependent, since nuclear localization of the fusion proteins was diminished when endogenous *Tnpo1* was downregulated (**S3 Fig**). In addition, mutation analyses showed that the TNPO1-promoted nuclear localization of the YFP fusion protein does not only depend on the presence of the PY-motif, but also of upstream basic residues that have been suggested to contribute to TNPO1 recognition [29] (**S4 Fig**). Together, these data are compatible with a role for TNPO1 in regulating the nuclear localization of the negative limb circadian clock proteins.

### PER proteins interact with TNPO1

**Fig 3:**
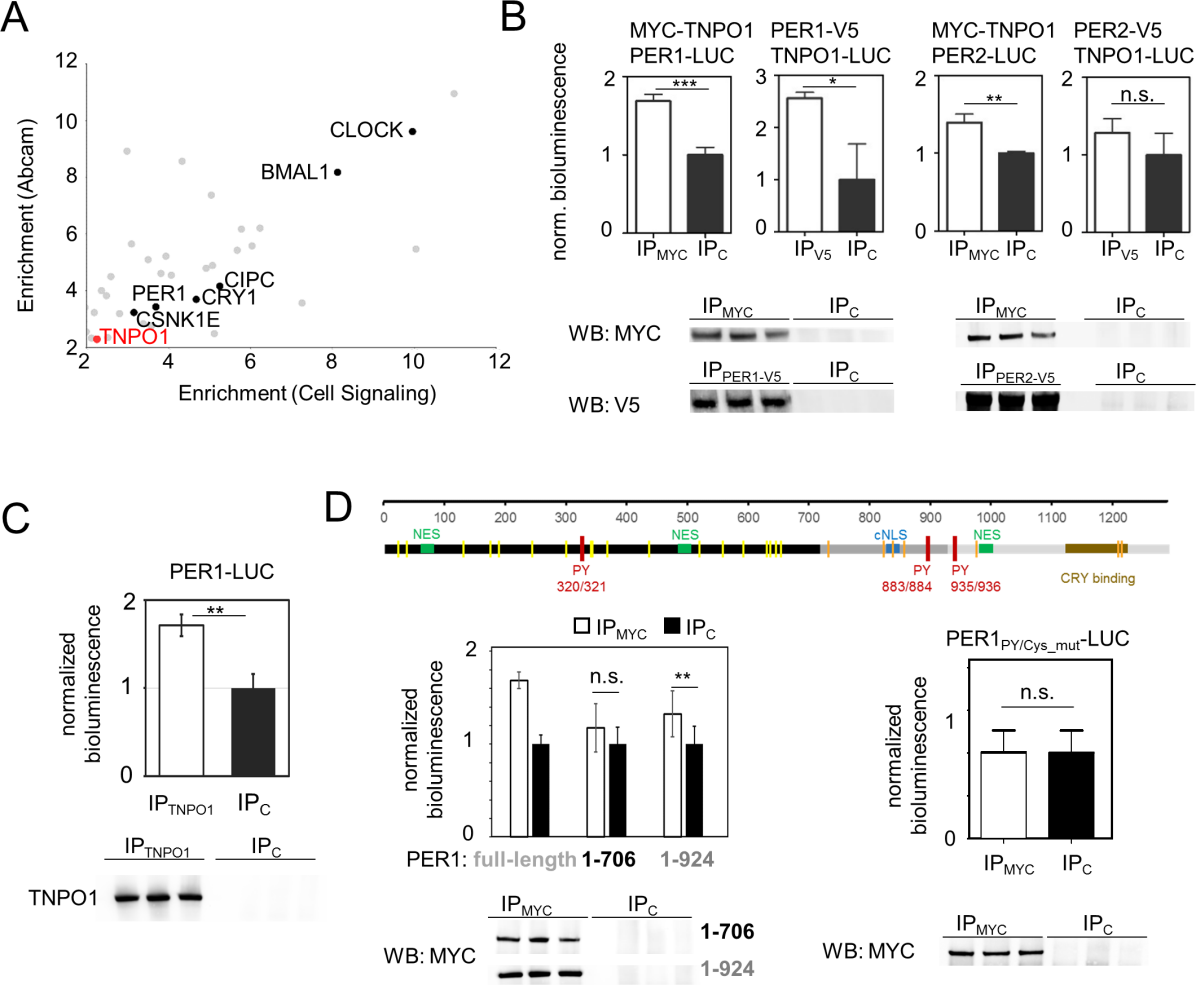
TNPO1 interacts with PER1. **(A)** Scatterplot of the results of two different anti-CLOCK immunoprecipitations (IPs) from unsynchronized U-2 OS cell nuclei followed by mass-spectrometry. Depicted is the enrichment of identified proteins relative to the starting cell lysate for two CLOCK-antibodies against each other after normalization to an IgG control IP (n = 3 independent IPs, Welch’s t-test). **(B)** Representative MYC- or V5-CoIPs (empty bars) normalized to IgG control IPs (filled bars) of HEK293 lysates (mean ± SD, n = 3 independent IPs, Student’s t-test: n.s. = non-significant; * p < 0.05; ** p < 0.01; *** p < 0.001). To control for efficient IP, western blots of either MYC-TNPO1 or PER1/2-V5 were performed using the specific anti-MYC or anti-V5 IPs as well as the unspecific control IgG IPs. Experiments were repeated two to seven times. (**C**) Co-immunoprecipitation from U-2 OS cells stably expressing a PER1-LUCIFERASE fusion protein with either an antibody targeting endogenous TNPO1 (IP_TNPO1_) or an IgG control (IP_C_). Shown are luciferase intensities of αTNPO1 IPs (empty bar) normalized to counts from IgG control IPs (filled bar). Given are means ± SD, n = 3 independent IPs (** p < 0.01, Student’s t-test). Representative western blots to control for efficient IPs are shown below. (**D**) TNPO1 interaction region is located in the C-terminal part of PER1. Top: Schematic illustration of the primary structure of human PER1. Shown are nuclear export sequences (NES, green), the classical nuclear localization sequence (cNLS, blue), the three PY-motifs (i.e. putative non-classical nuclear localization motifs, red), the CRY interaction domain (brown) as well as 25 cysteine residues that are conserved within human and mouse PER1 (yellow and orange). Different grey/black shades indicate the PER1 fragments used. Bottom left: Co-immunoprecipitation from HEK293 cells expressing MYC-TNPO1 and indicated fragments of PER1-LUCIFERASE with anti-MYC (empty bars) or control IgG (filled bars). For comparison, data for full-length PER1 are re-plotted from panel B. Given are means ± SD from nine independent IPs performed at three experimental days. One-way ANOVA with Bonferroni corrected posthoc test revealed indicated significance; ** p < 0.01; n.s. = non-significant). Controls for efficient IP are shown below. Bottom right: Co-immunoprecipitation from HEK293 cells expressing MYC-TNPO1 and a mutant version of PER1-LUCIFERASE (PER1_PY/Cys_mut_), in which both C-terminal PY-motifs (PY_883/834_ and PY_935/936_) as well as seven C-terminal cysteine residues (orange) are exchanged to alanine residues, with anti-MYC (empty bars) or control IgG (filled bars). Given are means ± SD of five independent immunoprecipitations. Representative western blots to control for efficient IPs are shown below.

To test whether TNPO1 interacts with endogenous circadian clock proteins, we analyzed nuclear circadian clock protein complexes in unsynchronized U-2 OS cells using immunoprecipitation with two different anti-CLOCK or control IgG antibodies followed by mass spectrometry. Indeed, endogenous TNPO1 was found to be part of such complexes together with known circadian clock proteins such as CLOCK, BMAL1, PER1, CRY1 and others (**Fig 3A**). To test for binding of TNPO1 to PER and CRY proteins, we performed co-immunoprecipitation experiments in HEK293 cells with epitope-tagged clock proteins but only detected very weak if any interaction signals (not shown). To increase the sensitivity of detecting also transient interactions, we performed a co-immunoprecipitation experiment using a luciferase-based readout. Since the putative TNPO1 recognition motifs of CRY proteins are not surface-accessible in the native protein [30, 31] we focused on PER proteins as established regulators of the negative feedback complex’s subcellular localization. MYC-tagged TNPO1 was immunoprecipitated from HEK293 cell lysates also containing PER1 or PER2 fused to full-length firefly luciferase. In addition, switching the orientation of the assay, we immunoprecipitated V5-tagged PER1 and PER2 and tested for TNPO1-luciferase binding. Only for PER1 a consistent and significant interaction with TNPO1 was detected in both orientations of the co-immunoprecipitation experiment (**Fig 3B**), while PER2 was only weakly detected in TNPO1-complexes (and *vice versa*). These results are further supported by luciferase complementation experiments where full-length PER proteins and TNPO1 were expressed as fusion proteins with firefly C- and N-terminal luciferase fragments in HEK293 cells [32]. Upon binding of PERs with TNPO1, a functional luciferase is reconstituted whose activity was measured in cell lysates. In this assay, PER1 and to a lesser extent (not significant) also PER2 but not the negative control βGAL promoted luciferase complementation (**S5 Fig**). Taken together, in both types of binding assays PER1 showed a robust interaction with TNPO1, while TNPO1 binding to PER2 was less reliably detectable suggesting that PER1 is a *bona fide* cargo of TNPO1. This is further supported by results from immunoprecipitation experiments using U-2 OS cells stably expressing a PER1-luciferase fusion protein, where we detected specific interaction with PER1 upon immunoprecipitation of endogenous TNPO1 (**Fig 3C**).

To investigate, which region of the PER1 sequence is responsible for TNPO1 interaction, we generated truncated versions of PER1, which lack either one or two C-terminal PY-motifs (**Fig 3D top**). While the shorter version (amino acids 1–706) did not specifically precipitate with TNPO1, the longer version (amino acids 1–924) was still significantly detectable in the TNPO1 precipitate, albeit with less intensity (**Fig 3D**) indicating that the C-terminal region of PER1 is required for TNPO1 binding. To test, whether these C-terminal PY-motifs and/or C-terminal cysteine residues (in analogy to the FOXO4-TNPO1 interaction [27]) are required for PER1-TNPO1 interaction, we generated a full-length, but mutant form of PER1, in which both PY-motifs and all seven cysteine residues that are conserved between mouse and human are exchanged by alanine residues (**Fig 3D top)**. Although the expression level of this mutant PER1 fusion protein was similarly high as wild-type (as estimated by the luciferase counts in the cell lysates), it did not specifically precipitate together with TNPO1 (**Fig 3D**) indicating that the C-terminal PY motifs and/or cysteine residues are required for PER1-TNPO1 interaction.

### Nuclear localization of PER1 is regulated by TNPO1

**Fig 4:**
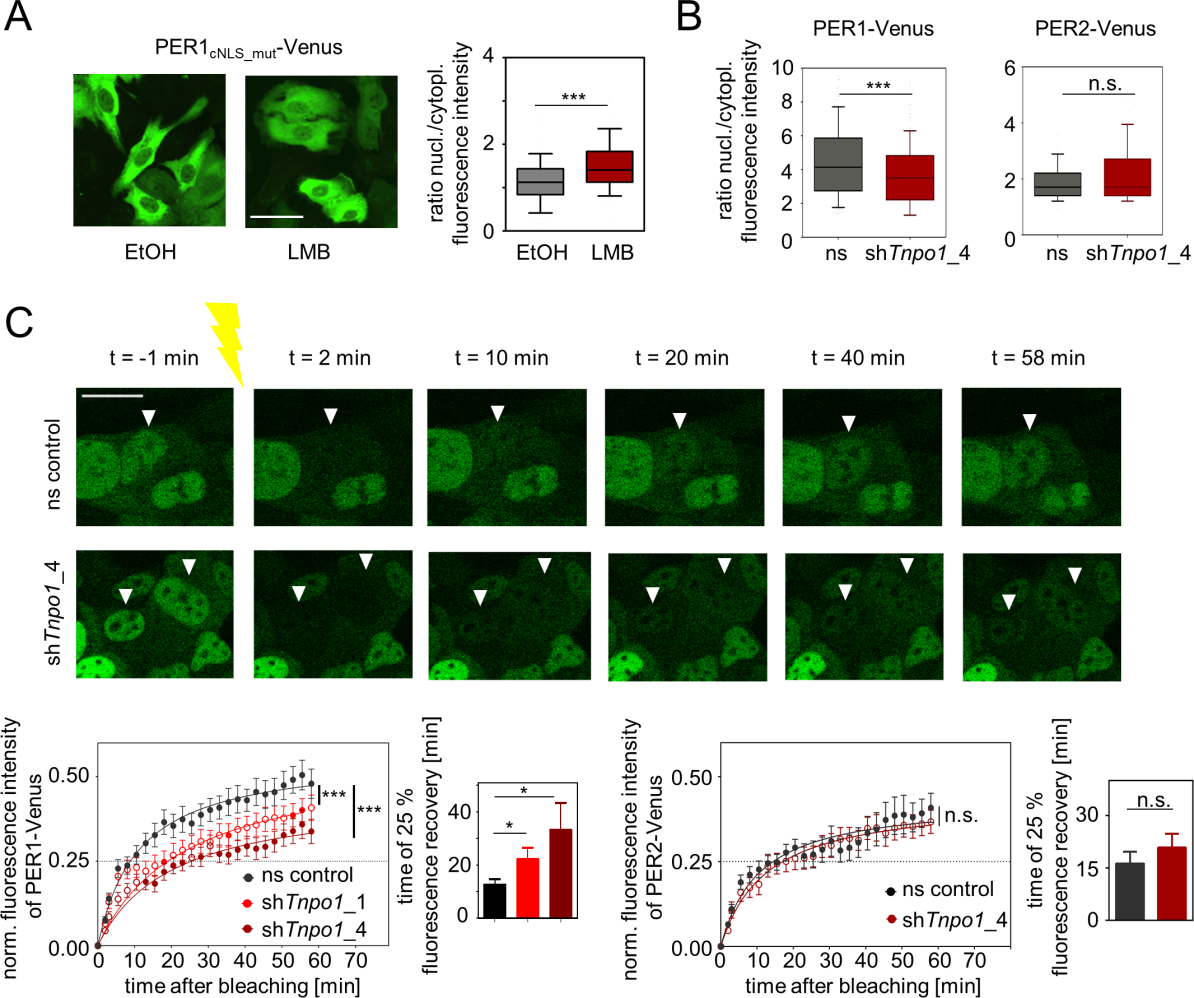
Nuclear localization of PER1 is regulated by TNPO1. (**A**) Depicted are representative fluorescence microscopic images (left) of U-2 OS cells ectopically expressing a version of PER1-Venus fusion protein, in which the classical NLS has been mutated (see also Fig 3D top). Cells were either treated with solvent (ethanol) or the nuclear export inhibitor Leptomycin B (LMB); scale bar: 50 μm. Right: Steady-state ratios of nuclear to cytoplasmic fluorescence intensity in single cells (box: median ± 25 percentile; whiskers: 10–90 percentile; n = 128 to 134 cells, statistics: Mann-Whitney-test; *** p < 0.001). **(B)** U-2 OS cells ectopically expressing PER1- or PER2-Venus fusion proteins were lentivirally transduced with shRNA constructs targeting *Tnpo1* or ns control shRNA. Depicted are steady-state ratios of nuclear to cytoplasmic fluorescence intensity in single cells (box: median ± 25 percentile; whiskers: 10–90 percentile; n = 247 to 744 cells, statistics: Mann-Whitney-test with Bonferroni-Holm posttest, n.s. = non-significant, *** q < 0.001). (**C)** Kinetics of nuclear import was analyzed using fluorescence recovery after photobleaching (FRAP) of individual nuclei of U-2 OS cells described in (B). Upper part: representative images with arrow heads indicating bleached nuclei (scale bar = 20 μm). For quantification (lower part) nuclear fluorescence intensity (normalized to cytoplasmic intensity) was determined per time point (left panels) and the average recovery time of 25% of initial fluorescence intensity was calculated (right panels). Given are means ± SEM, n = 8 to 18 individual cells. Statistics on import kinetics: two-way-ANOVA, posttest: Sidak‘s multiple comparison test, time points significantly different from ns control (p < 0.05) are indicated as filled circles. Statistics on 25% recovery time: Given are means ± SD, n = 10 to 17 individual cells, One-way ANOVA with Bonferroni-Holm corrected posthoc Student’s t-test (one-sided); * p < 0.05; *** p < 0.001, n.s. = non-significant.

It has been shown that the classical Importin α/β pathway has a dominant role for nuclear localization of PER proteins [20]; yet we still detected substantial nuclear staining of a PER1-Venus fusion protein, whose classical NLS has been mutated, which is further increased when nuclear export is pharmacologically inhibited (**Fig 4A**). While it is formally possible that nuclear localization of this mutant PER1 is mediated by endogenous interacting proteins that are shuttled *via* the Importin α/β-dependent pathway, we suggest that alternative nuclear transport pathways contribute to PER1 nuclear localization. To study whether TNPO1 acts as nuclear carrier for PER proteins, we analyzed the subcellular distribution of PER-Venus fusion proteins in the presence or absence of endogenous TNPO1 in unsynchronized U-2 OS cells. While the localization of PER2 is not altered upon *Tnpo1* depletion using RNAi, PER1’s localization is significantly less nuclear when TNPO1 is absent (**Fig 4B, S6 Fig**) suggesting that TNPO1 promotes PER1 but not PER2 nuclear translocation.

If TNPO1 is a nuclear import carrier for PER1, also the nuclear import kinetics should be decreased upon *Tnpo1* depletion. To test this, we performed fluorescence recovery after photobleaching (FRAP) experiments again with or without TNPO1. We bleached nuclear PER1-Venus in U-2 OS cells and imaged the recovery of fluorescence thereafter every 2.5 minutes, which largely corresponds to the nuclear import of PER1-Venus. Upon *Tnpo1* depletion (**S6 Fig**), the nuclear recovery of the PER1-Venus fluorescence is substantially slowed down with a mean 25% recovery time of 33 minutes in contrast to 13 minutes in controls (**Fig 4C, S1 and S2 Movies**). A less efficient *Tnpo1* knockdown construct resulted in an intermediate 25% recovery time of 22 minutes suggesting a dependence of PER1 nuclear import kinetics on TNPO1 expression levels. Such a correlation was not seen for PER2, where nuclear import kinetics was not significantly different when *Tnpo1* was depleted (**Fig 4C**).

### Oxidative stress strengthens PER1-TNPO1 binding decelerating PER1 nuclear import

**Fig 5:**
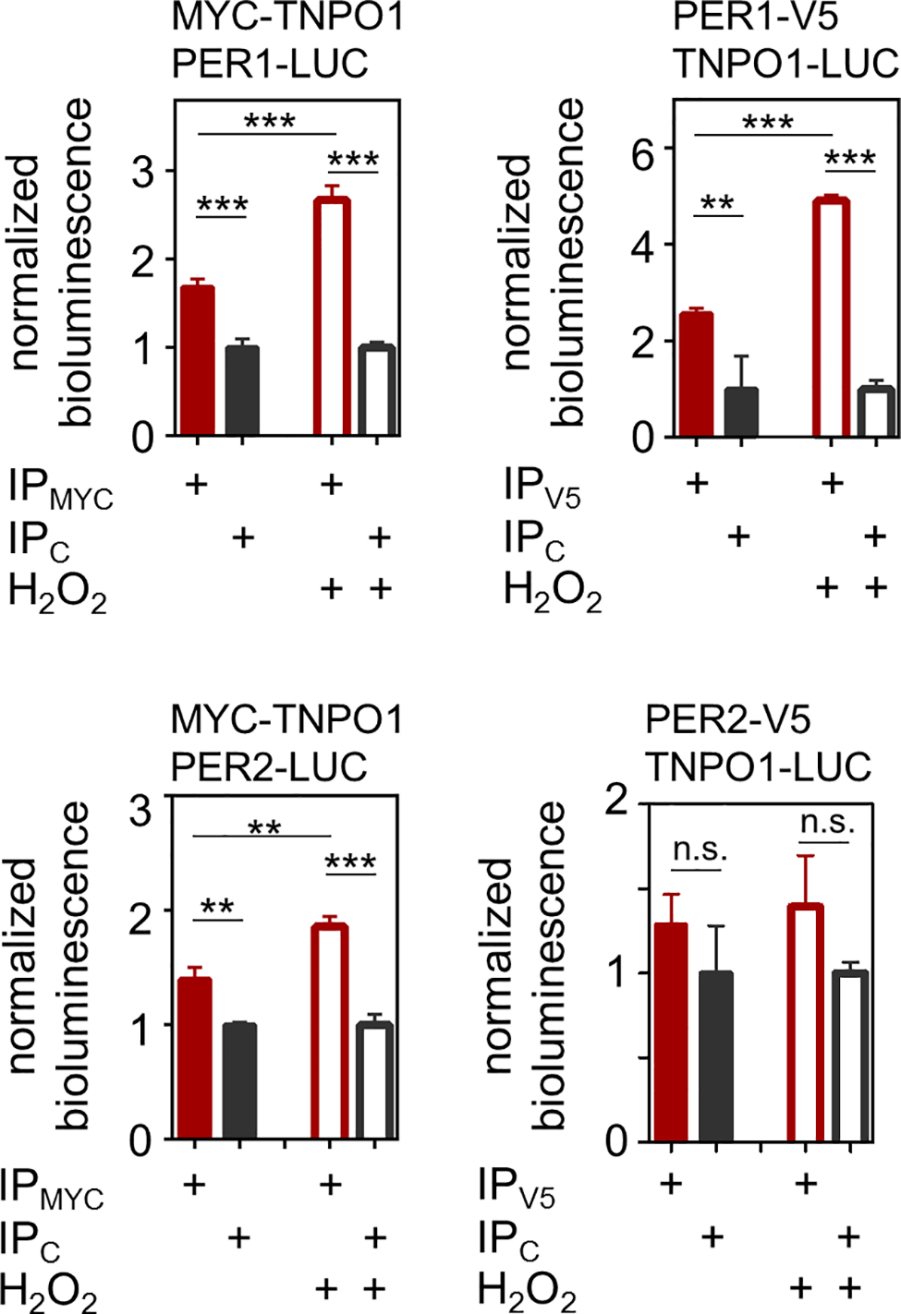
Oxidative stress leads to increased PER1-TNPO1 binding. Immunoprecipitation (IP) studies as described in Fig 3B were performed either under normal (filled bars) or oxidative stress (empty bars) conditions (200 μM H_2_O_2_). Shown are bioluminescence intensities upon MYC- or V5-CoIPs (red) normalized counts from IgG control IPs (black). Given are means ± SD, n = 3 independent IPs. One-way ANOVA revealed significant difference between columns (p < 0.001) except for the IP condition PER2-V5 TNPO1-LUC; Bonferroni post-test: n.s. = non-significant; * p < 0.05; ** p < 0.01; *** p < 0.001. Additional experiments gave similar results. For the IPs without H_2_O_2_ data from Fig 3B are re-plotted for comparison.

TNPO1-mediated nuclear transport has been described to be modulated by reactive oxygen species [27, 33]. For example, the nuclear import of the transcription factor FOXO4 is mediated via heterodimerization with TNPO1 through an intermolecular disulfide bond [27]. To test whether the TNPO1-PER1 interaction also responds to oxidative stress, we performed co-immunoprecipitation experiments after briefly treating cells or cell lysates with hydrogen peroxide (H_2_O_2_). Indeed, H_2_O_2_ treatment resulted in significantly higher interaction signals of PER1 (but not PER2) and TNPO1 (**Fig 5, S7 Fig**). This increase in interaction was H_2_O_2_ dose-dependent and also occurred with an alternative oxidizing reagent (diamide) but binding is abolished under reducing conditions (**S8 Fig**).

**Fig 6:**
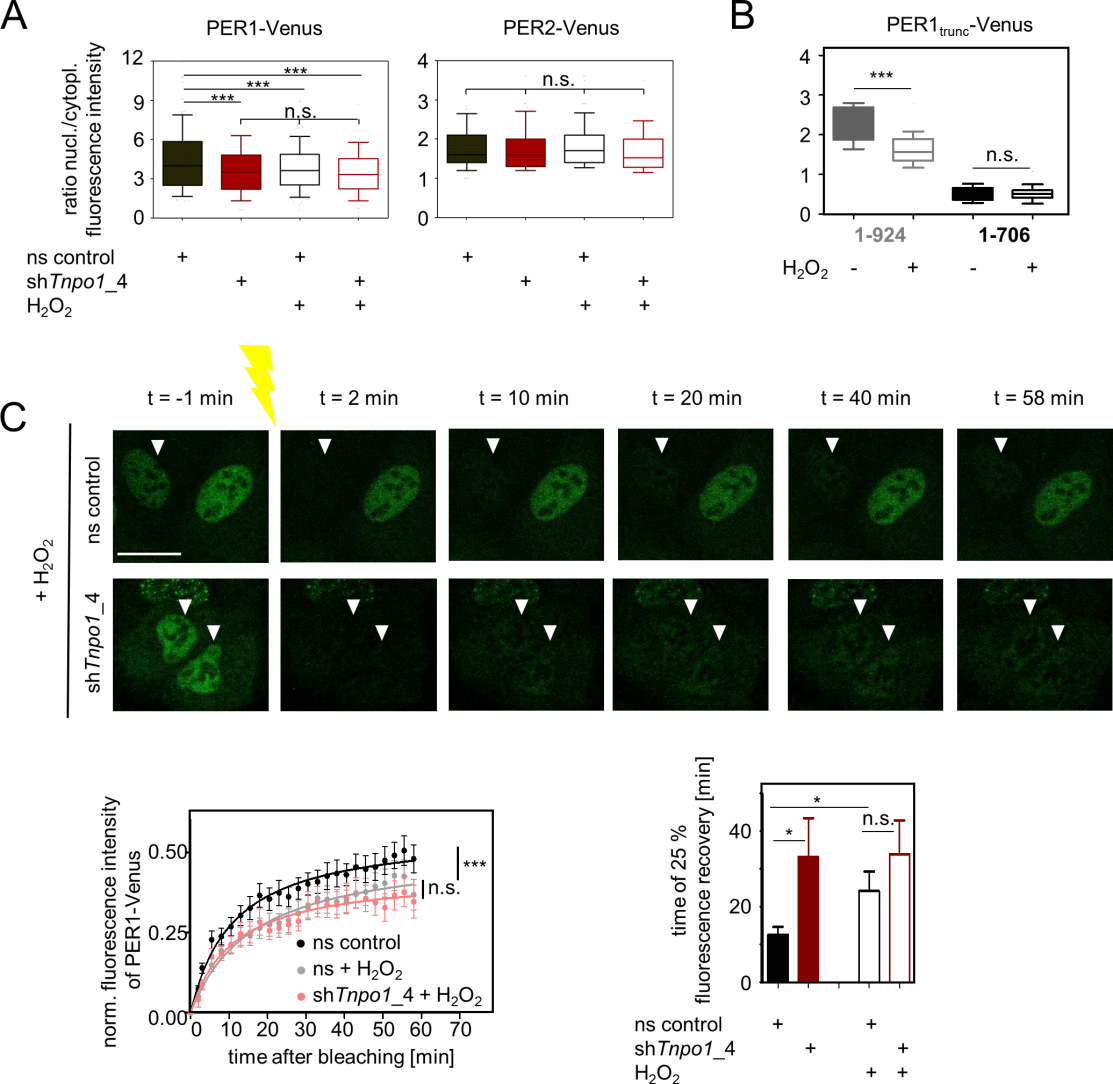
Nuclear localization is of PER1 is modulated by oxidative stress in a TNPO1-dependent manner. **(A)** Steady-state subcellular localization of ectopically expressed PER1- or PER2-Venus fusion proteins in TNPO1 depleted (red) or ns control (black) U-2 OS cells with or without H_2_O_2_ treatment. Depicted are nuclear to cytoplasmic fluorescence intensity ratios determined in four individually performed experiments (box: median ± 25 percentile; whiskers: 10–90 percentile, n = 185 to 801, One-way-ANOVA, posttest: Tukey’s multiple comparison test: n.s. = non-significant, *** p < 0.001). (**B**) Steady-state subcellular localization of ectopically expressed truncated versions (see also Fig 3D top) of PER1-Venus fusion proteins in U-2 OS cells with or without H_2_O_2_ treatment. Depicted are nuclear to cytoplasmic fluorescence intensity ratios (box: median ± 25 percentile; whiskers: 10–90 percentile, n = 21 to 59 cells, One-way-ANOVA, posttest: Tukey’s multiple comparison test: n.s. = non-significant, *** p < 0.001). **(C)** Kinetics of nuclear import upon H_2_O_2_ treatment was analyzed using fluorescence recovery after photobleaching (FRAP) of individual nuclei. Upper part: representative images with arrow heads indicating bleached nuclei (scale bar = 20 μm). Lower part, left: For quantification nuclear fluorescence intensity (normalized to cytoplasmic intensity) was determined per time point (left panels). Given are means ± SEM, n = 15 to 18 individual cells. For comparison, data for non-silencing control are re-plotted from Fig 4C. Two-way-ANOVA revealed indicated significance between treatment groups (*** p < 0.001, n.s. = non-significant). Lower part, right: The average recovery time of 25% of initial fluorescence intensity was calculated. Given are means of the times for 25% of initial PER1-Venus fluorescence recovery after photobleaching nuclei from *Tnpo1*depleted (red) or ns control (black) cells (error bars = SD, n = 10–17 cells for each condition, one-way ANOVA with Bonferroni-Holm corrected posthoc Student’s t-test (one-sided); * p < 0.05; n.s. = non-significant). Data of the two leftmost columns are re-plotted from Fig 4C.

Interestingly, increased binding upon H_2_O_2_ treatment did not lead to an increased nuclear import–on the contrary. When we measured subcellular localization of PER1-Venus in H_2_O_2_-treated cells, we observed significantly less nuclear PER1 (**Fig 6A**). This effect was TNPO1-dependent and specific for PER1, since H_2_O_2_-treatment neither affected PER1 localization in *Tnpo1*-depleted cells (**Fig 6A, S6 Fig**) nor did it alter PER2-Venus subcellular localization (**Fig 6A**). In addition, H_2_O_2_-treatment also affected the subcellular localization of the truncated version PER1_1-924_-Venus, but not the shorter PER1_1-706_-Venus version (**Fig 6B, S9 Fig**). Since the short PER1_1-706_ lacks the cNLS in addition to the two C-terminal PY-motifs and seven conserved cysteine residues (**Fig 3D top**), it is formally possible that any H_2_O_2_-mediated effect is undetectable due to the overall cytosolic localization of the fragment. Nevertheless, since PER1_1-706_ did not precipitate specifically with TNPO1 (see **Fig 3D**) these data may suggest that cysteine residues between amino acids 706 and 924 contribute to the H_2_O_2_-mediated alteration in subcellular localization of PER1. Furthermore, H_2_O_2_ decelerated the rate of nuclear PER1-Venus import measured in FRAP experiments but it had no effect on cells where *Tnpo1* is downregulated **(Fig 6C, S3 and S4 Movies)**. Together, this indicates that reactive oxygen species (ROS) modulate the nuclear import of PER1 by strengthening its interaction to TNPO1, yet in an unexpected direction–ROS slows down nuclear import of PER1.

## Discussion

Nuclear localization of circadian clock proteins at the right time of day–in particular of the negative limb–is crucial for circadian dynamics, since it defines the delay in negative feedback that determines the endogenous period. For example, in the nucleus PER and CRY proteins are thought to be almost exclusively present in a large 1.9 MDa complex. In the cytoplasm, however, PER and CRY proteins are incorporated into at least four (putative precursor) complexes of ∼0.9–1.1 MDa, two of which include PER1 but not PER2 [2]. Given the sizes of these complexes, active nuclear transport involving specialized import carriers is required for a directed and timely nuclear localization. Here, we show that nuclear shuttling of clock proteins (specifically PER1) is far more complex than previously assumed. Using systematic genetic perturbation of more than 60 components involved in nuclear-cytosolic translocation, we identified Transportin 1 (among others)—a non-classical nuclear import carrier—to be essential for a normal circadian period. TNPO1 interacts with PER1’s C-terminal region and is required for its timely nuclear localization, while TNPO1 depletion has no effect on PER2 localization.

Yet, both PER proteins also contain recognition motifs for the classical import mediated by the Importinα/β pathway that has been shown to be also critical for PER protein localization [20, 34, 35] as well as correct circadian period [25]. This implies that at least PER1 (and maybe also associated proteins) is transported to the nucleus by the classical pathway as well as the non-classical pathway via TNPO1. In fact, upon mutation of the classical NLS, the nuclear localization of PER1 is not completely abolished. It is not uncommon that one protein can be transported in the nucleus by more than one carrier [26]. Importantly, we do not exclude that TNPO1 has additional cargoes, whose timely transport is important for circadian dynamics. For example, although the PY-motifs found in CRY proteins are not surface-exposed in the native proteins, it is formally possible that CRYs bind to TNPO1 *via* their (reactive) cysteine residues that can occur in an oxidized state in cells [36].

It is intriguing that the effect on the circadian period upon knockdown of Importin β or TNPO1 goes in the opposite direction–long for Importin β depletion and short for TNPO1 depletion—although the depletion of both carriers leads to attenuated nuclear localization of PER1. This may be explained by the higher specificity of TNPO1 for primarily PER1-containing cytosolic sub-complexes [2], while Importin β probably contributes to shuttling of all PER/CRY complexes in the nucleus [25]. While the short period upon TNPO1 depletion is in agreement with the short period in wheel running behavior of *Per1* knockout mice [37], a mechanistic explanation is still difficult, since we do not fully understand, whether PER1 is the only TNPO1 cargo relevant for circadian dynamics, whether PER1-containing complexes are shuttled at different circadian times and with a different kinetics and whether (compared to PER2) PER1 has a fundamentally different role in the nucleus. We speculate that *Tnpo1* knockdown blocks an efficient nuclear transport of PER1 leading to an altered composition of the nuclear PER/CRY complex with more PER2 occupying the “PER slots” in the complex. We hypothesize that a complex with more PER2 has a higher repressive potential (which may explain the lower transcript levels of *Per* genes upon *Tnpo1* knockdown). This is in agreement with recent data from the Weitz lab [2] showing that loss of PER1 or PER2 differentially affects the actions of CK1δ within the nuclear PER complex, which might have an effect on its repressive power. An alternative hypothesis may be that *Tnpo1* knockdown allows PER1 to incorporate into the PER/CRY complexes more rapidly leading to a faster “maturation” and shuttling of the nuclear complex, since the competition between Importin β-mediated and TNPO1-mediated nuclear translocation is shifted towards the presumably faster Importin β-mediated shuttling. This might lead to both a more efficient transcriptional repression (reflected by lower transcript levels of *Per* genes) and a shorter period.

In recent years, it became increasingly obvious that cellular redox state and the canonical transcriptional-translational circadian clock are intimately linked (for a review, see [38]). Yet, while it is well accepted that redox state is under circadian regulation and feeds back to the clock (also as possible adaptation to redox stress) the underlying molecular mechanisms are very poorly understood. Here, we identify an additional link between redox state and circadian clock by showing that TNPO1-PER1 interaction is strengthened upon oxidative stress—very similar to the effects observed for other TNPO1 cargoes, i.e. FOXO4 [27] and DJ-1 [33]. Whether this increased binding occurs via disulfide bonds (as for the FOXO-TNPO1 interaction) is currently unclear, but it is conceivable given the reactive cysteine residues described for PER proteins [36]. In fact, both truncation of the C-terminal ~500 amino acids of PER1 that contain two PY-motifs and seven conserved cysteine residues as well as mutation of all these residues abolished TNPO1 binding. Since for PER2 C-terminal cysteine residues (amino acids 1210 and 1213 of mPER2) have been implicated in CRY1 binding [36], it is possible that also for PER1 such C-terminal cysteine residues are relevant for CRY binding. Further work is needed to pinpoint the exact location and characterize a potentially differential role of the putative reactive cysteine residues in PER1.

Surprisingly, however, increased binding to TNPO1 upon oxidative stress did not lead to accelerated nuclear import–on the contrary, import was slowed down. This was very specific for PER1 and TNPO1, since firstly PER2 import rate was unaffected by redox stress and secondly redox stress had not effect on PER1 import kinetics when TNPO1 was depleted. We can only speculate, why increased binding of PER1-TNPO1 leads to slower import (similar to *Tnpo1* knockdown): We probably observe in FRAP experiments the combined effect of Importin β and TNPO1 on PER1 import rate. Assuming that Importin β-mediated transport is faster than TNPO1-mediated transport, a stronger binding to TNPO1 might shift the relative contribution of the two carriers towards the slower one. Preventing PER1 nuclear localization in oxidative conditions might be a mechanism to boost the expression of the antioxidant defense master regulator *Nrf2*, a CLOCK/BMAL1 target gene that can it is inhibited by PER/CRY complex in the nucleus [39]. In addition, PER1 has been assigned an anti-apoptotic role [40, 41], thus a reduced nuclear localization of PER1 in oxidative conditions might be protective through promoting apoptosis. Future experiments are required to unravel the mechanisms and impact of TNPO1-mediated redox crosstalk to the clock.

Apart from TNPO1, we identified several additional players associated with nuclear-cytoplasmic translocation to be important for circadian dynamics. Again, knockdown of such components resulted in period changes with opposite directions even if they seem to be involved in similar processes. For example, knockdown of nuclear pore complex proteins NUP160, NUP153 or NUP85 led to long and knockdown of NUP54 to short periods for yet unknown reasons. Similarly, depletion of nuclear export carrier XPO1 (also known as Exportin 1) resulted in long period, while knockdown of RanBP16 (also known as Exportin 7) in short periods. The genes *kpnb1*, *xpo1*, *sec13*, which show a circadian phenotype upon knockdown in our screen, have been previously suggested to alter circadian dynamics upon knockdown or inhibition. KPNB1 mediates PER/CRY nuclear translocation and is required for a normal circadian clock function [25]. Pharmacological inhibition of XPO1 has been shown to lengthen the circadian period in an inhibitor dose-dependent manner [14]. In addition, the nucleo-cytoplasmic translocation protein SEC13 was identified as an essential component for normal circadian rhythm generation [42]. Different members of the kapα-family, KPNA1, KPNA3 and KPNA7 have been shown to bind to wild type CRY2 but not a cNLS mutant of the circadian clock protein [21]. In line with those findings, altered PER1/PER2 localization upon misregulation of *kpna2* expression was reported [24]. However, we did not find alterations upon knockdown of individual members of the *kapα* family members, which may be due to redundancy or compensation effects by other members of this gene family. In general, in screening endeavors, negative results need to be carefully interpreted, because of such effects and because knockdown efficiency cannot be evaluated for every single shRNA construct.

Together, these data emphasize our lack in knowledge about how the cell achieves timely subcellular localization of clock protein complexes and its associated consequences for circadian dynamics. We predict several levels of complexity: (i) clock proteins have more than one carrier, e.g. PER1 (and associated proteins) is transported by Importin β and TNPO1; (ii) transport processes might be regulated in a time-dependent manner; e.g. *Tnpo1* transcript levels are rhythmic (almost in-phase with *Per1*) and anti-phasic to *Importin β* transcript rhythms [43, 44]; (iii) signaling may have an impact on the relative usage of the alternative carriers; e.g. redox stress increases interaction of PER1 with TNPO1 and slows down its nuclear import. Considering a similar complexity for nuclear export processes of both clock proteins and clock mRNAs, it becomes obvious that much more work is needed to unravel such regulatory mechanisms. Therefore, the work presented here identifying TNPO1 as carrier for PER1, its role for circadian dynamics as well as its regulation by cellular redox state is a first step in this direction.

## Materials and methods

### RNAi screen

RNAi constructs were purchased from Open Biosystems. Lentiviruses were produced in HEK293T cells in a 96-well plate format essentially as described [45]. Virus-containing supernatants were filtered and U-2 OS (human, American Type Culture Collection [ATCC] # HTB-96) reporter cells were transduced with 100 μl of virus filtrate plus 8 ng/μl protamine sulfate. After 1 d, medium was exchanged to puromycin-containing (10 μg/ml) medium prior to bioluminescence recording.

### Bioluminescence recordings

U-2 OS cells (human, ATCC HTB-96) stably expressing firefly luciferase from a *Bmal1* promoter fragment [11] were seeded either onto a white 96-well plate (2×104 cells/well) or in 30 mm NUNC dishes (2x105cells/well). After 72 hours, cells were synchronized with dexamethasone (1 μM) for 30 minutes, washed with PBS and cultured in Phenol-Red-free DMEM containing 10% fetal bovine serum, antibiotics (100 U/ml penicillin and 100 μg/ml streptomycin) and 250 μM D-luciferin (Biothema, Darmstadt, Germany). Bioluminescence recordings were performed at 35–37°C in a 96-well plate luminometers (TopCount, PerkinElmer, Rodgau, Germany) or LumiCycle (Actimetrics, Düsseldorf, Germany). Data were analyzed using ChronoStar software as described previously [11].

### CRISPR/Cas9

The generation and validation of U-2 OS TNPO1 knockout cells was performed as previously reported for FBXL3 knockout U-2 OS cells [46]. Briefly: Oligonucleotides specific for the target site of *Tnpo1* were designed using the Optimized CRISPR Design tool (http://crispr.mit.edu/) and ligated into the lentiCRISPR v2 plasmid (Addgene #52961) [47] using a BsmBI restriction site. PCR-products for sequencing were phosphorylated and ligated into a pUC19 vector. Single clones were sequenced using the M13 forward primer.

### Quantitative RT-PCR

Total RNA was prepared using Pure Link RNA Mini Kit (Life Technologies) according to the manufacturer’s protocol and then reversely transcribed to cDNA using M-MLV Reverse Transcriptase (Life Technologies). Quantitative PCR was performed with SYBRGreen fluorescence assays and analyzed in a CFX96 machine (Bio-Rad, Munich, Germany). For quantitative PCR, QuantiTect primers (Qiagen) were used except for Gapdh (hGAPDH_fwd: TGCACCACCAACTGCTTAGC, hGAPDH_rev: ACAGTCTTCTGGGTGGCAGTG). The transcript levels were normalized to Gapdh and evaluated according to the 2-ddCt method.

### Western blot

Western blotting was performed essentially as described [11]. Briefly, cells were harvested in RIPA lysis buffer containing 1:100 protease inhibitor cocktail (Sigma, Munich, Germany) or PKB lysis buffer [27] without protease inhibitors. Equal amounts of protein were separated by SDS-PAGE using 4% to 12% Bis-Tris gels (Life Technologies), transferred to nitrocellulose membrane, and incubated over night with anti-TNPO1 antibody (1:1000, ab10303, Abcam), anti-βACTIN (1:100,000, A3853, Sigma) anti-V5 (R960-25, Invitrogen) or anti MYC-antibody (sc40, Santa Cruz Technologies). Next day, membranes were probed with HRP-conjugated secondary antibodies (donkey anti rabbit (sc2305, Santa Cruz Technologies) or goat anti mouse (sc2005, Santa Cruz Technologies), 1:1000 in TBST), and a chemiluminescence assay was performed using Super Signal West Pico substrate (Pierce, Rockford, IL) followed by protein detection.

### Subcellular distribution

For subcellular distribution assay of PY-peptides, corresponding sequences were PCR-amplified from U-2 OS wild type cDNA and ligated into pEYFP or pECFP (Clontech) using BglII and SalI restriction sites. RNAi constructs, harboring a GFP, were mutated to generate an out-of-frame shift and thus non-fluorescent GFP expression vectors. Fluorescence imaging was performed using either the Leica DMIL LED Fluo fluorescence microscope, the LeicaDM6000 confocal microscope Sp5 or the Olympus IX81 confocal microscope and the Leica Application Suite software, V3.7 or the Olympus Fluoview software. Image analysis were performed using ImageJ 1.44p.

### Chromatin immunoprecipitation and mass spectrometry

We performed a modified (unpublished) standard chromatin immunoprecipitation (ChIP) in triplicates using 1 mg of formaldehyde cross-linked protein extracts from unsynchronized U-2 OS with the following antibodies: rabbit anti-CLOCK (Abcam), rabbit anti-CLOCK (Cell Signaling) and rabbit anti-IgG (Cell Signaling). After the final wash of the ChIP, proteins bound to ChIP-grade agarose beads (Cell Signaling) were digested into peptides as follows. First, 50 μl of digestion buffer (2 M urea in 50 mM Tris, pH 7.5, 2 mM DTT and 1 μg of trypsin) was added and incubated for 30 min at 37°C. Subsequently, beads were spin down and the supernatants collected and saved. Then, another 50 μl of 2 M urea in 50 mM Tris, pH 7.5, and 10 mM chloroacetamide was added to the beads prior to incubation at 37°C for 5 min. Beads were then spin down and the supernatants were collected and combined with those saved from the previous step. The supernatant mixture was then incubated over-night at 25°C to complete protein digestion that was stopped in the morning by adding 1 μl trifluoroacetic acid. Peptides were then cleaned for MS measurement using SDB-RPS stage tips as described [48]. Half of the peptide volume was used for the analysis using an LC 1200 ultra-high-pressure system (Thermo Fisher Scientific) coupled via a nano-electrospray ion source (Thermo Fisher Scientific) to a Q Exactive HF Orbitrap (Thermo Fisher Scientific). Prior to MS, the peptides were separated on a 50 cm reversed-phase column (diameter of 75 mm packed in-house with ReproSil-Pur C18-AQ 1.9 mm resin [Dr. Maisch GmbH]) over a 120 min gradient of 5%-60% buffer B (0.1% formic acid and 80% ACN). Full MS scans were acquired in the 300–1,650 m/z range (R = 60,000 at 200 m/z) at a target of 3e6 ions. The fifteen most intense ions were isolated, fragmented with higher-energy collisional dissociation (HCD) (target 1e5 ions, maximum injection time 120 ms, isolation window 1.4 m/z, NCE 27%, and underfill ratio of 20%), and finally detected in the Orbitrap (R = 15,000 at 200 m/z).

### MS data processing and statistical analysis

The raw MS files were processed using MaxQuant (version 1.5.5.2) with the integrated Andromeda search engine using FDR < 0.01 [49]. Variable modifications for oxidized methionine (M) and acetylation (protein N-term) as well as a fixed modification for carbamidomethyl (C) were included in the search. The standard “match between runs” option was enabled. For the identification of peptides and proteins the UniProt human FASTA database (from September 2014) was used. Bioinformatics analyses were performed with the Perseus software (version 1.5.4.1) [50]. After removing potential contaminants as well as reverse sequences, label free intensities were transformed to logarithm with base 2 and entries that contained less than 2 values in at least one group (IgG control, CLOCK (Abcam) or CLOCK (Cell Signaling)) were filtered out. Missing values from the remaining proteins were imputed using random values from the normal intensity distribution with a down shift (2.3) and a width 0.3 (as described in [51]. We then performed a Welch’s t-test using the triplicate label free intensities of each protein in the IgG (control) versus either CLOCK precipitates.

### Co-immunoprecipitation (CoIP)

For CoIP assays full-length coding sequences (or mutant/truncated versions) of PER1, PER2 and TNPO1 were cloned into Gateway destination vectors (pcDNADest40-V5, pEFDest51-Luc, pcMYC-CMV-D12) according to the manufacturer’s protocol (Invitrogen, Darmstadt, Germany). HEK293 cells were grown to 60% confluence, transfected with equal amounts of DNA with Lipofectamine2000 (Thermo Fisher Scientific) according to the manufacturer's protocol. 48 hours after transfection, cells coexpressing either PER1/2-V5 and TNPO1-Luc or PER1/2-Luc and MYC-TNPO1 were harvested in PKB buffer [27] without any protease and phosphatase inhibitor cocktails. To generate a U-2 OS cell line stably expressing PER1-LUCIFERASE, the full-length coding sequences of PER1 was cloned in to a pLenti6 vector with the CDS of firefly luciferase at the C-terminus. Also for these cells, harvest of cell lysate was performed using PKB buffer [27] without any protease and phosphatase inhibitor cocktails. About 500 μl of whole cell lysates (corresponding to 1 to 4 mg total protein) were pre-cleared with 30 μl of agarose G+ beads at 4°C for one hour on a rotating wheel. Lysates were centrifuged at 3500 rpm at 4°C for 5 minutes. Supernatant was transferred to low-binding tubes and 2 μg of either anti-TNPO1 (ab10303, Abcam), anti-MYC (sc-40, Santa Cruz Technologies), anti-V5 (R960-25, Invitrogen) or normal mouse IgG (sc-2025, Santa Cruz Technologies) antibody were added and incubated for one hour on a rotating wheel prior to washing and luminescence measurement. For determination of bioluminescence, the CoIPs were washed two to four times with ice cold 1 x PBS and dried with a 27G x 0.75” syringe. Repeated measurements using the β-scout device (PerkinElmer) were performed up to 10 minutes. Luminescence counts were analyzed relative to the counts of the normal mouse IgG control CoIPs.

### Nuclear import kinetics

For the expression of Venus-fusion proteins, either full-length or mutant/truncated versions of mPER2 CDS or mPER1 CDS were shuttled into a pLenti6 vector with the CDS of the fluorophore at the C-terminus [32]. Confocal microscopy of live cells was performed with an Olympus IX81 microscope (Olympus, Tokyo, Japan) with a ×60 (1.35 numerical aperture) water-immersion objective in a climate chamber at 37°C under 5% CO_2_. Dynamics of nuclear import were measured by bleaching nuclear fluorescence of cells expressing Venus-tagged versions of PER1 or PER2. Recovery of fluorescence was observed by taking pictures every 2.5 minutes. Mean nuclear and cytoplasmic fluorescence was calculated and mean background fluorescence was subtracted. Initial nuclear fluorescence was set to 1.0 and the bleached fraction was set to 100% [52]. Nuclear recovery was normalized to changes in cytoplasmic fluorescence to compensate for overall bleaching due to repeated measurements.

### Pharmacological treatment

The nuclear export inhibitor leptomycin B (LMB) was added 60 min before imaging at a final concentration of 10 ng/ml. Unless indicated, induction of oxidative stress was performed using 200 μM hydrogen peroxide (or 200 μM diamide) for 30 minutes prior to measurements and imaging. For the luciferase-based CoIPs H_2_O_2_ was added approximately every hour from the time point of cell lysis till measurement of luminescence. To induce reducing conditions, Tris(2-carboxyethyl)phosphine (TCEP) was added to PKB lysis buffer at a final concentration of 1 mM. Notably, the H_2_O_2_ concentration used here is much higher than measured under physiological or pathophysiological conditions, in which concentrations usually do not exceed the low-micromolar range. However, exogenously added H_2_O_2_ rapidly degrades by cellular catalase and peroxidases, thus it is very common in bolus-based experiments that H_2_O_2_ concentrations in the upper micromolar or even millimolar range are used to evoke a cellular response [for a review see [53]].

### Luciferase complementation assay

The luciferase complementation assay was performed as described in Kucera et al., 2012 [32]. Briefly: CRY1, βGal, PER1/2 and TNPO1 CDS were cloned into pcdnaDest40-Luc or Luc-pEFDest51 using the Gateway Cloning system. HEK293 cells were transiently transfected with a pair of split firefly luciferase reporter construct (400 ng each transfection). For normalization, the renilla luciferase vector pRL-SV40 (4 ng; Promega, Mannheim, Germany) was cotransfected. 48 hours after transfection cells were lysed in 200 μL passive lysis buffer (Promega,) and frozen for at least one hour at −80°C. The Dual-Luciferase Reporter Assay System (Promega) and a multisample plate-reading luminometer (Orion II, Berthold Detection Systems) was used to measure luciferase activity of the cell lysates.

### Statistics

Statistics was performed using GraphPad Prism version 5.00 for Windows (GraphPad Software, La Jolla California USA).

## Supporting information

S1 Fig Knockdown of *Tnpo1* alters clock gene expression. Transcript rhythms of indicated genes in dexamethasone-synchronized U-2 OS cells that were transduced either with a shRNA targeting *Tnpo1* (red curves) or a non-silencing control hairpin (black curves). Data are normalized to *Gapdh* expression and presented relative to mean expression in control cells. Shown are mean ± SEM levels of three independent samples (except for five time points, where there were only two samples).
(PDF)

S2 Fig CRISPR/Cas9 generated TNPO1 knockout single cell clones. **(A)** Sequencing results of the two *Tnpo1* alleles of single cell clones after limited dilution of the gRNA1 population. Out-of-frame-shifts are indicated in red, whereas in-frame-shifts are depicted in green. **(B)** Representative *Bmal1*-luciferase oscillations of a single TNPO1 knockout cell clone (1_1) or single wild type (wt) cell clones. **(C)** Left: mean period of independent bioluminescence recordings (error bars = SD, n = 7; Student‘s t-test: ** p < 0.01). Right: representative western blot of TNPO1 and beta-ACTIN protein levels in genome-edited (1_1) or wild type (wt) cells.
(PDF)

S3 Fig PER and CRY proteins contain functional TNPO1 recognition motifs. Clock protein-derived peptides harboring a PY-containing putative TNPO1 recognition motif were fused to CFP and expressed in either TNPO1-depleted or control HEK293 and U-2 OS cells for analysis of subcellular localization. The hnRNPA1 M9 peptide was used as positive control. (**A)** Boxplot of the normalized nuclear to cytoplasmic fluorescence intensity ratios of cells described in (A). The ratio was normalized to the mean of the negative control of each individual experiment. Box: median ± 25 percentile; whiskers: 5–95 percentile, n = 23–187 cells. Statistics: Mann-Whitney-test with Bonferroni-Holm posttest, a: PY-peptide in ns cells compared to CFP only in ns cells (q < 0.001); b and c: PY-peptide in KD cells (all cells either transduced with sh*Tnpo1*_1 or sh*Tnpo1*_4) compared to ns cells (b: q < 0.001, c: q < 0.05). (**B**) Representative residual TNPO1 protein level in HEK293 or U-2 OS cell lysates after transduction of RNAi knocking down *Tnpo1* expression (sh*Tnpo1*_1 or sh*Tnpo1*_4) or control shRNA (ns control). These data serve as knockdown controls for data shown in (A).
(PDF)

S4 Fig Nuclear localization of clock protein-derived PY-peptides depends on TNPO1 and critical residues. Schematic illustration of mutated versions of the YFP-CRY2 (246–289) fusion that were transfected into U-2 OS cells for subcellular localization analysis. YFP-BMAL1 (533–581) was used as negative control (see S3B Fig). Box: median ± 25 percentile; whiskers: 10-90 percentile; n = 54-71 cells per condition; statistics: Mann-Whitney-test with Bonferroni-Holm posttest, a: compared to CRY2wt peptide, b: compared to BMAL1 peptide, *** p < 0.001; scale bar = 50 μm.
(PDF)

S5 Fig TNPO1 interacts with PER1. HEK293 cells were cotransfected with renilla luciferase and firefly luciferase fragments (LUC1 or LUC2) fused to indicated proteins. LUC1 (the N-terminal fragment of luciferase) was fused N-terminally to TNPO1 (LUC1-TNPO1), whereas LUC2 (the C-terminal fragment of luciferase) was C-terminally fused to either PER1 (dark red, PER1-LUC2) or PER2 (red, PER2-LUC2). Upon binding of PERs with TNPO1, a functional luciferase is reconstituted whose activity was measured in cell lysates. CRY1 binding to PER proteins served as positive controls (green) and LUC2-*β*GAL as negative control (grey). Reconstituted firefly luciferase activity was normalized on full length renilla luciferase activity. Depicted is one out of three experiment (error bars = SD, n = 3 individually transfected measurements, one-way ANOVA with Dunnett’s posttest: n.s. = non-significant, * p < 0.05, ** p < 0.01, *** p < 0.001). Two additional experiments gave similar results.
(PDF)

S6 Fig Knockdown efficiency of shRNA constructs targeting *Tnpo1*. Residual mRNA (top) and protein (bottom) level of TNPO1 after transduction of U-2 OS cells with RNAi targeting either *Tnpo1* expression (sh*Tnpo1*_1 and sh*Tnpo1*_4, red) or ns control shRNA (black). These data serve as knockdown controls for data shown in Figs 4 and 6.
(PDF)

S7 Fig Immunoprecipitation efficiencies. To control for efficient immunoprecipitation upon co-immunoprecipitation of TNPO1 and PER1/2 under oxidative stress, western blots of either MYC-TNPO or PER1/2-V5 were performed using the specific anti-MYC or anti-V5 IPs as well as the unspecific control IgG IPs. Experiments were repeated two to five times with similar results. These data serve as controls for data shown in Fig 5.
(PDF)

S8 Fig Oxidative stress leads to increased PER1-TNPO1 binding. **(A)** TNPO1 interaction with PER1 is H_2_O_2_-dose dependent. Co-immunoprecipitation (IP) from U-2 OS cells stably expressing a PER1-LUCIFERASE fusion protein with either an antibody targeting endogenous TNPO1 (IP_TNPO1_) or an IgG control (IP_C_) using indicated concentrations of H_2_O_2_. Shown are luciferase intensities of αTNPO1 IPs (red) normalized to counts from IgG control IPs (black). Given are means ± SD, n = 3 independent IPs. Two-way ANOVA revealed a significant effect of antibody type (p < 0.001) and H_2_O_2_ concentration (p < 0.005). Representative western blots to control for efficient IPs are shown below. (**B**) TNPO1 interaction with PER1 is dependent on oxidizing conditions. Co-immunoprecipitation from U-2 OS cells stably expressing a PER1-LUCIFERASE fusion protein with either an antibody targeting endogenous TNPO1 (αTNPO1) or an IgG control using oxidizing (200 μM diamide) or reducing (1 mM TCEP) conditions. Shown are luciferase intensities of αTNPO1 IPs (red) normalized to counts from IgG control IPs (black). Given are means ± SD, n = 3–4 independent IPs. Student’s t-test revealed a significant difference between diamide or TCEP treatment compared to solvent (* p < 0.05). Representative western blots to control for efficient IPs are shown below.
(PDF)

S9 Fig Oxidative stress leads to altered PER1 nuclear localization. Steady-state subcellular localization of ectopically expressed truncated versions of PER1-Venus fusion proteins in U-2 OS cells with or without H_2_O_2_ treatment (scale bar 20 μm). Quantification see Fig 6A and 6B.
(PDF)

S1 Table Circadian periods upon RNAi-knockdown of all tested genes. Period deviations (mean ± SD) compared to non-silencing controls (ns) and significances for each individual construct (Dunnett’s posttest) as well as the Holm-Bonferroni corrected q value for each gene is given. Bold: Genes, for which at least two different RNAi constructs were used, resulted in significantly altered period in the same direction.
(PDF)

S2 Table Putative M9 sequences of clock proteins. Clock protein-derived sequences containing at least one evolutionary conserved PY motif.
(PDF)

S1 Movie FRAP control. Representative fluorescence recovery time-lapse recordings of PER1-VENUS fusion proteins in U-2 OS ns control cells.
(AVI)

S2 Movie FRAP *Tnpo1* knockdown. Representative fluorescence recovery time-lapse recordings of PER1-VENUS fusion proteins in *Tnpo1* knockdown U-2 OS cells.
(AVI)

S3 Movie FRAP control plus H_2_O_2_. Representative fluorescence recovery time-lapse recordings of PER1-VENUS fusion proteins in U-2 OS ns control cells treated with H_2_O_2_.
(AVI)

S4 Movie FRAP *Tnpo1* knockdown plus H_2_O_2_. Representative fluorescence recovery time-lapse recordings of PER1-VENUS fusion proteins in *Tnpo1* knockdown U-2 OS cells treated with H_2_O_2_.
(AVI)
