# Supplementary figures and images for: The non-classical nuclear import carrier Transportin 1 modulates circadian rhythms through its effect on PER1 nuclear localization
Source: PLoS Genet. 2018 Jan 29;14(1):e1007189. doi: 10.1371/journal.pgen.1007189 (PMC5805371; doi:10.1371/journal.pgen.1007189)

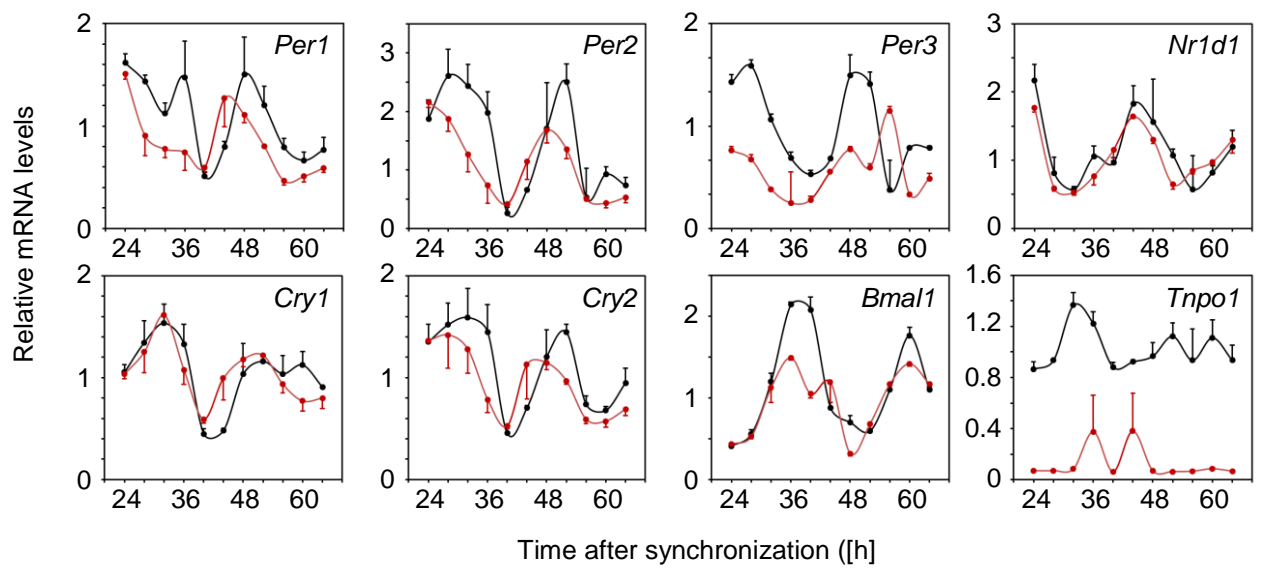

Supplement: S1 Fig — Transcript rhythms of indicated genes in dexamethasone-synchronized U-2 OS cells that were transduced either with a shRNA targeting Tnpo1 (red curves) or a non-silencing control hairpin (black curves). Data are normalized to Gapdh expression and presented relative to mean expression in control cells. Shown are mean ± SEM levels of three independent samples (except for five time points, where there were only two samples). (PDF) [file pgen.1007189.s001.pdf]

**A**

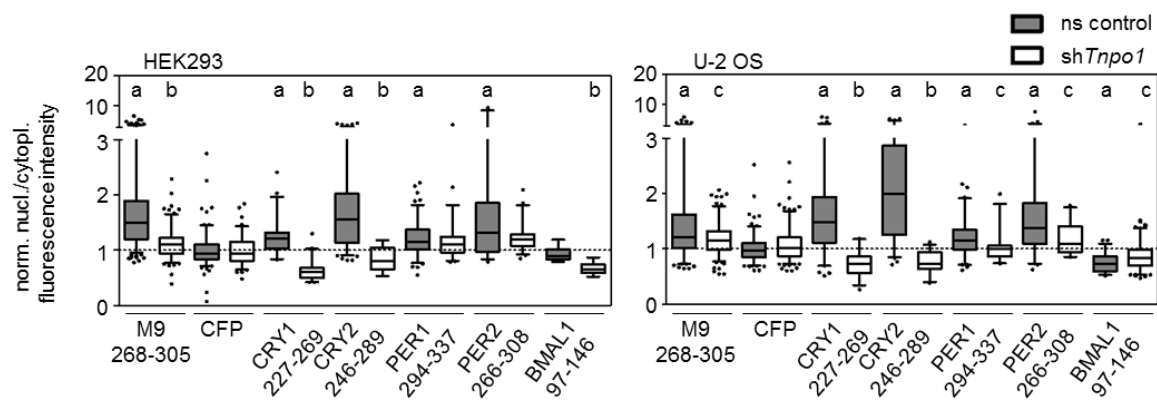

**B**

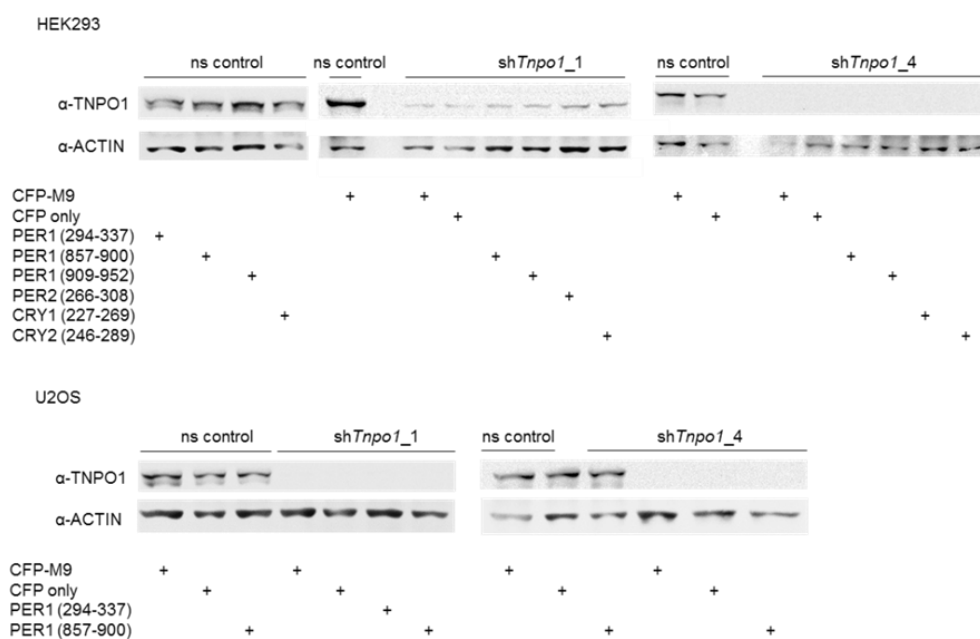

Supplement: S3 Fig — Clock protein-derived peptides harboring a PY-containing putative TNPO1 recognition motif were fused to CFP and expressed in either TNPO1-depleted or control HEK293 and U-2 OS cells for analysis of subcellular localization. The hnRNPA1 M9 peptide was used as positive control. (A) Boxplot of the normalized nuclear to cytoplasmic fluorescence intensity ratios of cells described in (A). The ratio was normalized to the mean of the negative control of each individual experiment. Box: median ± 25 percentile; whiskers: 5–95 percentile, n = 23–187 cells. Statistics: Mann-Whitney-test with Bonferroni-Holm posttest, a: PY-peptide in ns cells compared to CFP only in ns cells (q < 0.001); b and c: PY-peptide in KD cells (all cells either transduced with shTnpo1_1 or shTnpo1_4) compared to ns cells (b: q < 0.001, c: q < 0.05). (B) Representative residual TNPO1 protein level in HEK293 or U-2 OS cell lysates after transduction of RNAi knocking down Tnpo1 expression (shTnpo1_1 or shTnpo1_4) or control shRNA (ns control). These data serve as knockdown controls for data shown in (A). (PDF) [file pgen.1007189.s003.pdf]

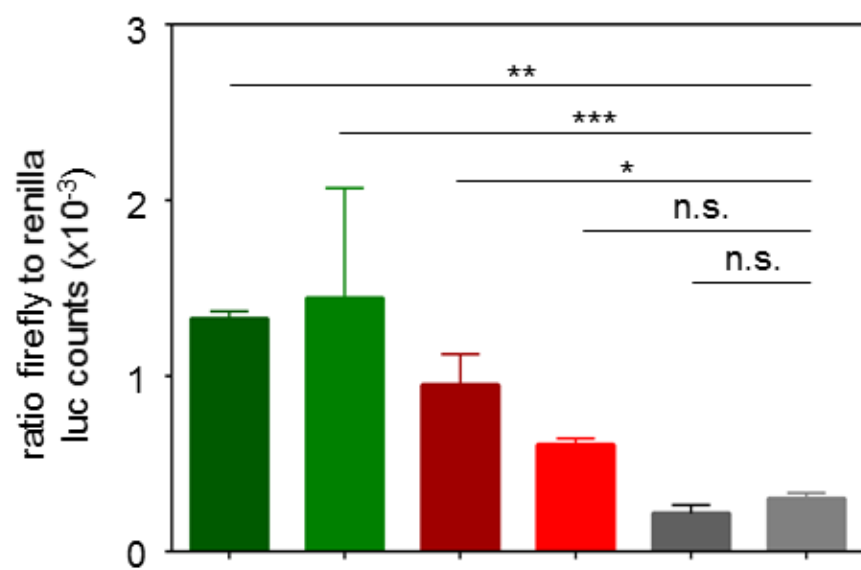

LUC2-β-Gal  
CRY1-LUC2  
LUC1-PER1  
PER1-LUC2  
PER2-LUC1  
PER2-LUC2  
LUC1-TNPO1

+ + + + + +  
+ + + + + +  
+ + + + + +  
+ + + + + +  
+ + + + + +  
+ + + + + +  
+ + + + + +

Supplement: S5 Fig — HEK293 cells were cotransfected with renilla luciferase and firefly luciferase fragments (LUC1 or LUC2) fused to indicated proteins. LUC1 (the N-terminal fragment of luciferase) was fused N-terminally to TNPO1 (LUC1-TNPO1), whereas LUC2 (the C-terminal fragment of luciferase) was C-terminally fused to either PER1 (dark red, PER1-LUC2) or PER2 (red, PER2-LUC2). Upon binding of PERs with TNPO1, a functional luciferase is reconstituted whose activity was measured in cell lysates. CRY1 binding to PER proteins served as positive controls (green) and LUC2-βGAL as negative control (grey). Reconstituted firefly luciferase activity was normalized on full length renilla luciferase activity. Depicted is one out of three experiment (error bars = SD, n = 3 individually transfected measurements, one-way ANOVA with Dunnett’s posttest: n.s. = non-significant, * p < 0.05, ** p < 0.01, *** p < 0.001). Two additional experiments gave similar results. (PDF) [file pgen.1007189.s005.pdf]

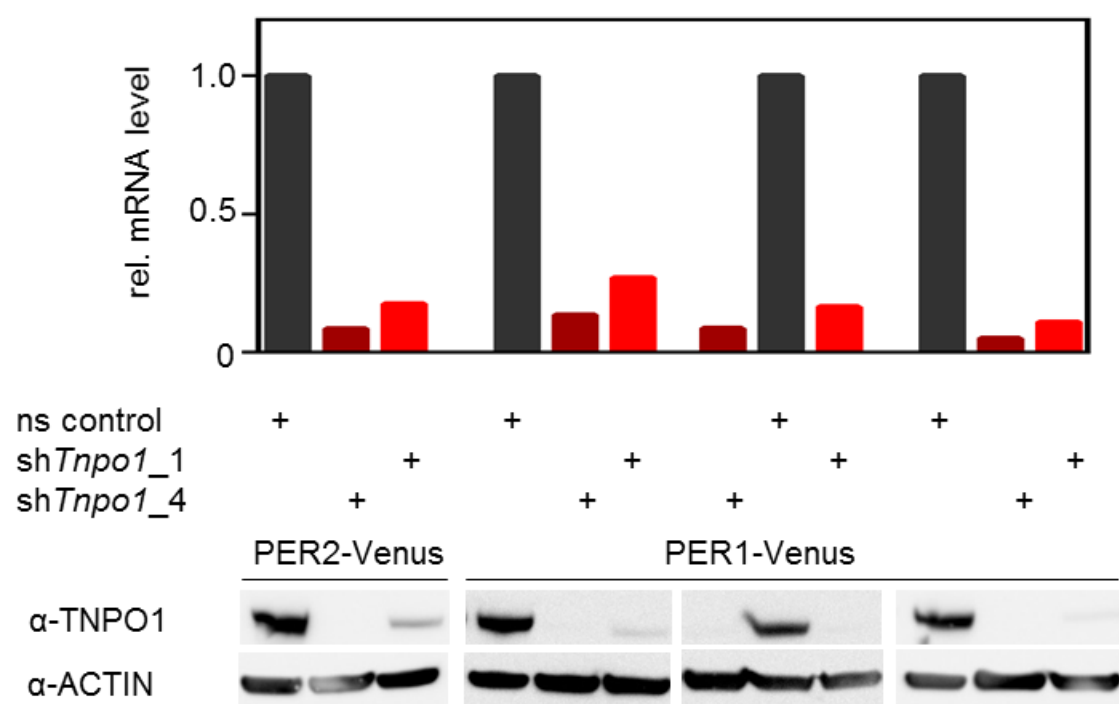

Supplement: S6 Fig — Residual mRNA (top) and protein (bottom) level of TNPO1 after transduction of U-2 OS cells with RNAi targeting either Tnpo1 expression (shTnpo1_1 and shTnpo1_4, red) or ns control shRNA (black). These data serve as knockdown controls for data shown in Figs 4 and 6. (PDF) [file pgen.1007189.s006.pdf]

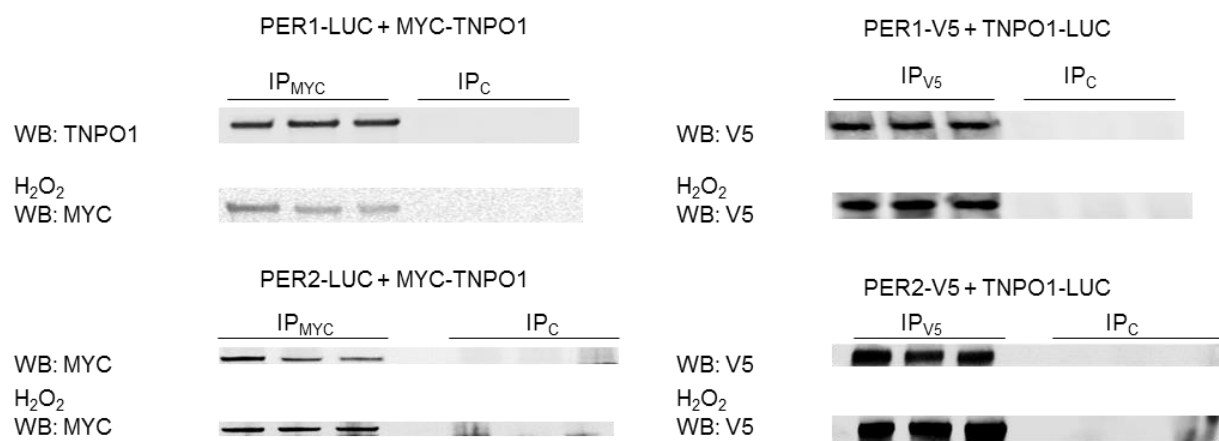

Supplement: S7 Fig — To control for efficient immunoprecipitation upon co-immunoprecipitation of TNPO1 and PER1/2 under oxidative stress, western blots of either MYC-TNPO or PER1/2-V5 were performed using the specific anti-MYC or anti-V5 IPs as well as the unspecific control IgG IPs. Experiments were repeated two to five times with similar results. These data serve as controls for data shown in Fig 5. (PDF) [file pgen.1007189.s007.pdf]

A

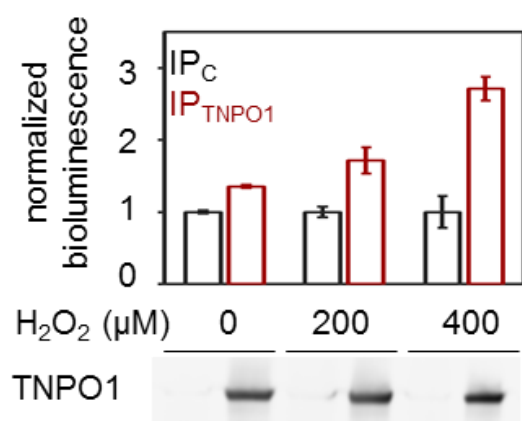

B

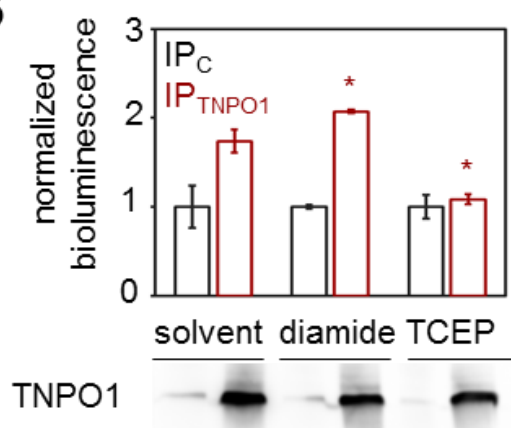

Supplement: S8 Fig — (A) TNPO1 interaction with PER1 is H2O2-dose dependent. Co-immunoprecipitation (IP) from U-2 OS cells stably expressing a PER1-LUCIFERASE fusion protein with either an antibody targeting endogenous TNPO1 (IPTNPO1) or an IgG control (IPC) using indicated concentrations of H2O2. Shown are luciferase intensities of αTNPO1 IPs (red) normalized to counts from IgG control IPs (black). Given are means ± SD, n = 3 independent IPs. Two-way ANOVA revealed a significant effect of antibody type (p < 0.001) and H2O2 concentration (p < 0.005). Representative western blots to control for efficient IPs are shown below. (B) TNPO1 interaction with PER1 is dependent on oxidizing conditions. Co-immunoprecipitation from U-2 OS cells stably expressing a PER1-LUCIFERASE fusion protein with either an antibody targeting endogenous TNPO1 (αTNPO1) or an IgG control using oxidizing (200 μM diamide) or reducing (1 mM TCEP) conditions. Shown are luciferase intensities of αTNPO1 IPs (red) normalized to counts from IgG control IPs (black). Given are means ± SD, n = 3–4 independent IPs. Student’s t-test revealed a significant difference between diamide or TCEP treatment compared to solvent (* p < 0.05). Representative western blots to control for efficient IPs are shown below. (PDF) [file pgen.1007189.s008.pdf]

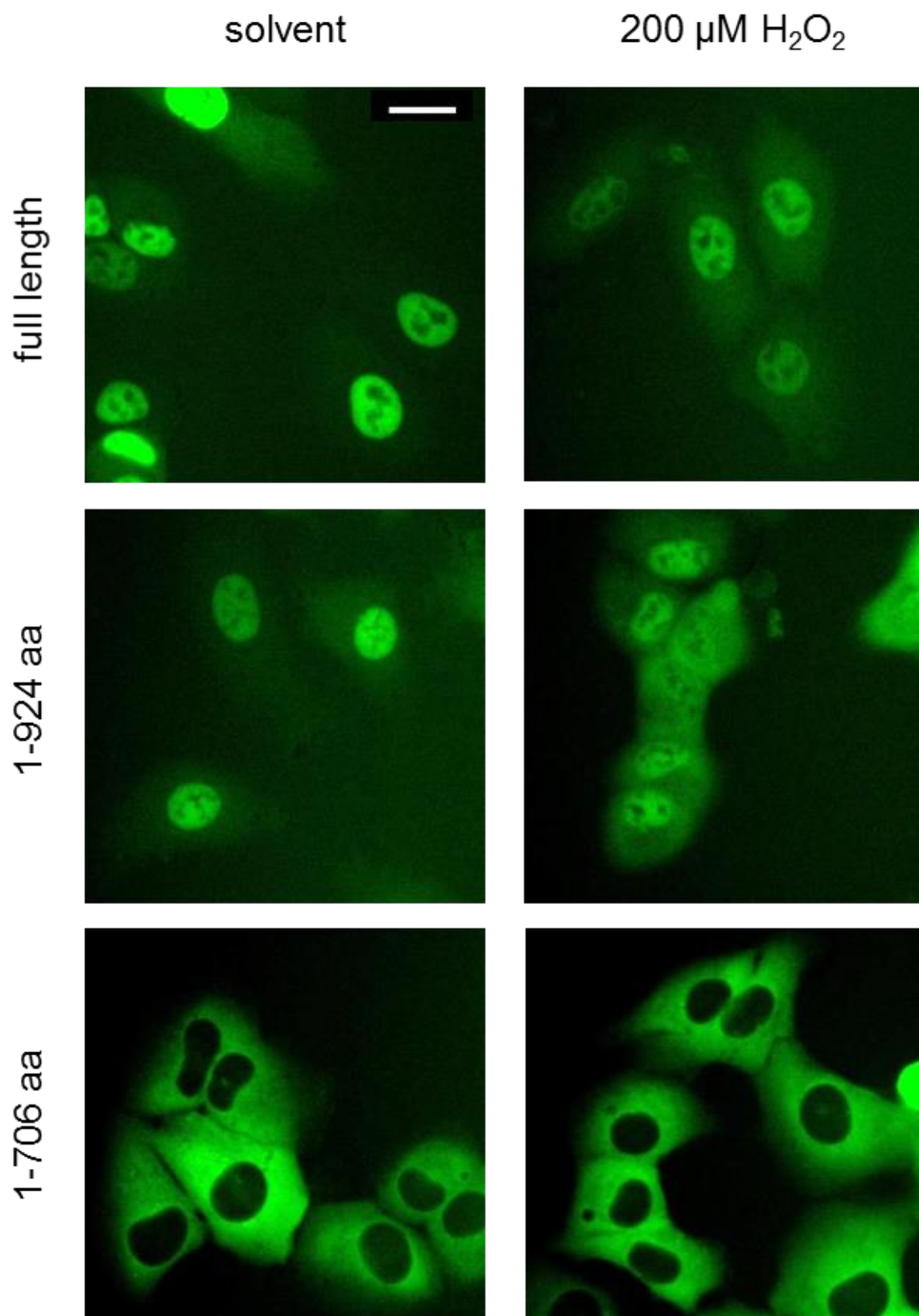

Supplement: S9 Fig — Steady-state subcellular localization of ectopically expressed truncated versions of PER1-Venus fusion proteins in U-2 OS cells with or without H2O2 treatment (scale bar 20 μm). Quantification see Fig 6A and 6B. (PDF) [file pgen.1007189.s009.pdf]
