# Supplementary material for: The non-classical nuclear import carrier Transportin 1 modulates circadian rhythms through its effect on PER1 nuclear localization
Source: PLoS Genet. 2018 Jan 29;14(1):e1007189. doi: 10.1371/journal.pgen.1007189 (PMC5805371; doi:10.1371/journal.pgen.1007189)
Supplement: S2 Fig — (A) Sequencing results of the two Tnpo1 alleles of single cell clones after limited dilution of the gRNA1 population. Out-of-frame-shifts are indicated in red, whereas in-frame-shifts are depicted in green. (B) Representative Bmal1-luciferase oscillations of a single TNPO1 knockout cell clone (1_1) or single wild type (wt) cell clones. (C) Left: mean period of independent bioluminescence recordings (error bars = SD, n = 7; Student‘s t-test: ** p < 0.01). Right: representative western blot of TNPO1 and beta-ACTIN protein levels in genome-edited (1_1) or wild type (wt) cells. (PDF) [file pgen.1007189.s002.pdf]

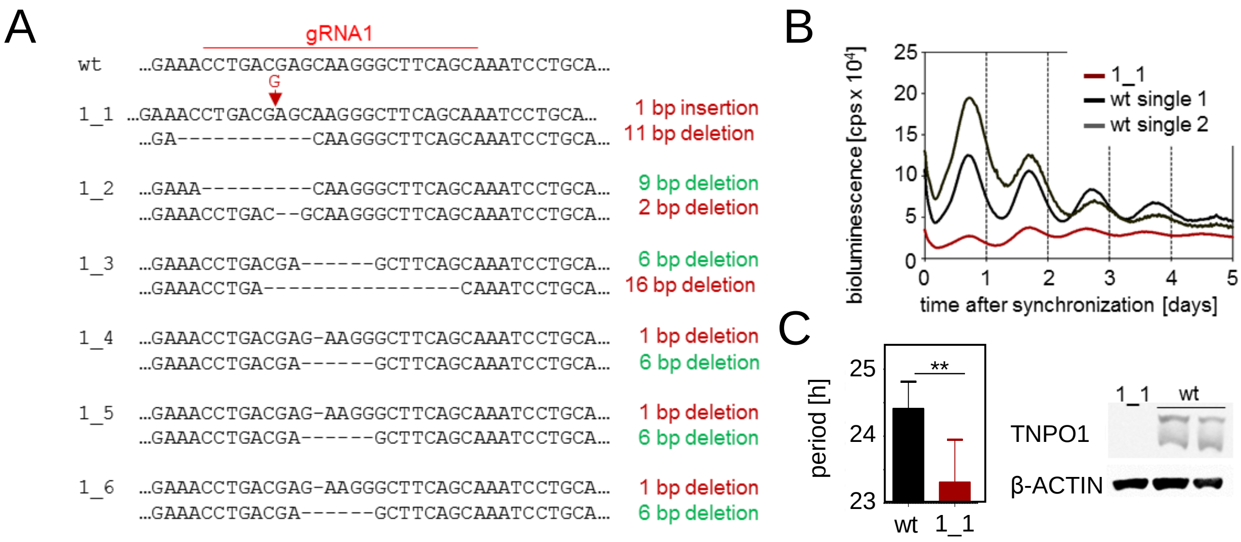
A
gRNA1
wt ...GAAACCTGACGAGCAAGGGCTTCAGCAAATCCTGCA...
G
1_1 ...GAAACCTGACGAGCAAGGGCTTCAGCAAATCCTGCA... 1 bp insertion
...GA-----------CAAGGGCTTCAGCAAATCCTGCA... 11 bp deletion
1_2 ...GAAA---------CAAGGGCTTCAGCAAATCCTGCA... 9 bp deletion
...GAAACCTGAC--GCAAGGGCTTCAGCAAATCCTGCA... 2 bp deletion
1_3 ...GAAACCTGACGA------GCTTCAGCAAATCCTGCA... 6 bp deletion
...GAAACCTGA----------------CAAATCCTGCA... 16 bp deletion
1_4 ...GAAACCTGACGAG-AAGGGCTTCAGCAAATCCTGCA... 1 bp deletion
...GAAACCTGACGA------GCTTCAGCAAATCCTGCA... 6 bp deletion
1_5 ...GAAACCTGACGAG-AAGGGCTTCAGCAAATCCTGCA... 1 bp deletion
...GAAACCTGACGA------GCTTCAGCAAATCCTGCA... 6 bp deletion
1_6 ...GAAACCTGACGAG-AAGGGCTTCAGCAAATCCTGCA... 1 bp deletion
...GAAACCTGACGA------GCTTCAGCAAATCCTGCA... 6 bp deletion
B
bioluminescence [cps x 10^4]
25
20
15
10
5
0
1_1
wt single 1
wt single 2
0 1 2 3 4 5
time after synchronization [days]
C
period [h]
25
24
23
**
wt 1_1
1_1 wt
TNPO1
β-ACTIN
