# Supplementary material for: The non-classical nuclear import carrier Transportin 1 modulates circadian rhythms through its effect on PER1 nuclear localization
Source: PLoS Genet. 2018 Jan 29;14(1):e1007189. doi: 10.1371/journal.pgen.1007189 (PMC5805371; doi:10.1371/journal.pgen.1007189)
Supplement: S4 Fig — Schematic illustration of mutated versions of the YFP-CRY2 (246–289) fusion that were transfected into U-2 OS cells for subcellular localization analysis. YFP-BMAL1 (533–581) was used as negative control (see S3B Fig). Box: median ± 25 percentile; whiskers: 10-90 percentile; n = 54-71 cells per condition; statistics: Mann-Whitney-test with Bonferroni-Holm posttest, a: compared to CRY2wt peptide, b: compared to BMAL1 peptide, *** p < 0.001; scale bar = 50 μm. (PDF) [file pgen.1007189.s004.pdf]

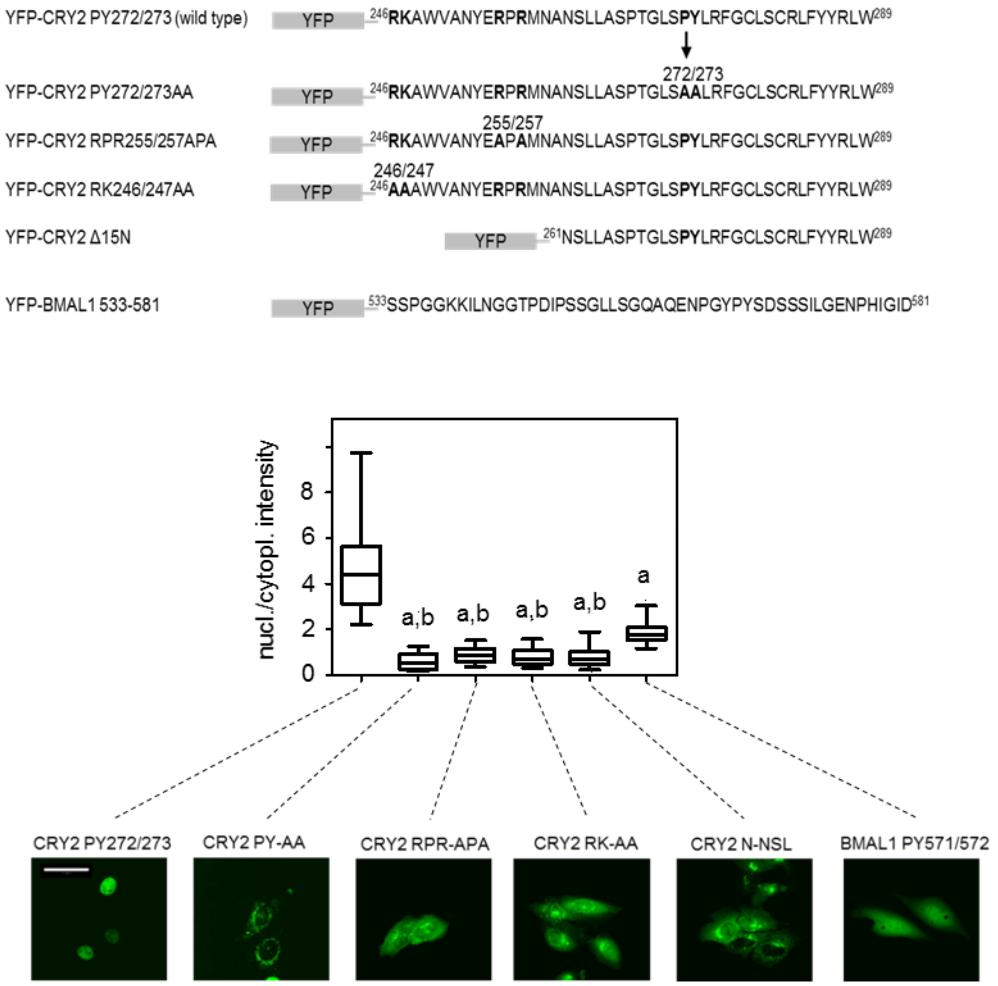
YFP-CRY2 PY272/273 (wild type)
YFP
246RKAWVANYERPRMNANSLLASPTGLSPYLRFGCLSCRLFYYRLW289
272/273
YFP-CRY2 PY272/273AA
YFP
246RKAWVANYERPRMNANSLLASPTGLSAALRFGCLSCRLFYYRLW289
255/257
YFP-CRY2 RPR255/257APA
YFP
246RKAWVANYEAPAMNANSLLASPTGLSPYLRFGCLSCRLFYYRLW289
246/247
YFP-CRY2 RK246/247AA
YFP
246AAAWVANYERPRMNANSLLASPTGLSPYLRFGCLSCRLFYYRLW289
YFP-CRY2 Δ15N
YFP
261NSLLASPTGLSPYLRFGCLSCRLFYYRLW289
YFP-BMAL1 533-581
YFP
533SSPGGKKILNGGTPDIPSSGLLSGQAQENPGYPYSDSSSILGENPHIGID581
nucl./cytopl. intensity
0
2
4
6
8
a,b
a,b
a,b
a,b
a
CRY2 PY272/273
CRY2 PY-AA
CRY2 RPR-APA
CRY2 RK-AA
CRY2 N-NSL
BMAL1 PY571/572
