# Supplementary material for: The non-classical nuclear import carrier Transportin 1 modulates circadian rhythms through its effect on PER1 nuclear localization
Source: PLoS Genet. 2018 Jan 29;14(1):e1007189. doi: 10.1371/journal.pgen.1007189 (PMC5805371; doi:10.1371/journal.pgen.1007189)
Supplement: S1 Table — Period deviations (mean ± SD) compared to non-silencing controls (ns) and significances for each individual construct (Dunnett’s posttest) as well as the Holm-Bonferroni corrected q value for each gene is given. Bold: Genes, for which at least two different RNAi constructs were used, resulted in significantly altered period in the same direction. (PDF) [file pgen.1007189.s010.pdf]

Target gene	q value	RNAi construct 1			RNAi construct 2			RNAi construct 3		
		Dunnetts posttest	mean period deviation [h]	SD	Dunnetts posttest	mean period deviation [h]	SD	Dunnetts posttest	mean period deviation [h]	SD
ns control			0.0	0.2						
fbxl3	9E-38	***	3.6	0.2	***	3.4	0.1	***	2.1	0.2
sec13	**2E-34**	*******	**3.2**	**0.1**	*******	**2.6**	**0.4**	*******	**1.7**	**0.2**
nup205	4E-27	***	3.3	0.6	n.s.	0.3	0.2	n.s.	-0.2	0.2
nup160	**1E-26**	*******	**2.3**	**0.3**	*******	**1.8**	**0.1**			
kpnb1	**2E-23**	*******	**2.8**	**1.1**	*******	**2.2**	**0.7**	**n.s.**	**0.0**	**0.3**
nup98	7E-22	***	2.1	0.2	***	0.6	0.2	*	-0.3	0.1
tnpo1	**2E-20**	*******	**-1.8**	**0.1**	*******	**-1.1**	**0.2**	*******	**-0.8**	**0.0**
elys	**1E-17**	*******	**1.2**	**0.1**	*******	**0.9**	**0.1**	*******	**0.6**	**0.1**
seh1	**2E-17**	*******	**1.6**	**0.1**	*******	**0.5**	**0.1**	******	**0.4**	**0.1**
ranbp10	4E-14	***	1.3	0.1	***	-0.7	0.5	n.s.	-0.3	0.1
nup155	2E-13	***	0.7	0.1	*	-0.3	0.1	n.s.	0.0	0.1
ranbp11	**3E-12**	*******	**-0.8**	**0.2**	*******	**-0.7**	**0.1**	**n.s.**	**-0.1**	**0.3**
ranbp16	**9E-12**	*******	**-1.0**	**0.1**	*******	**-0.6**	**0.1**	**n.s.**	**0.1**	**0.1**
ranbp2	4E-11	***	-0.7	0.3	***	0.6	0.2	***	0.6	0.3
nup133	9E-11	***	1.1	0.1	*	-0.4	0.1	n.s.	-0.2	0.2
nup153	**3E-10**	*******	**1.3**	**0.0**	*******	**1.1**	**0.0**	**n.s.**	**0.3**	**0.1**
gle1	**1E-08**	*******	**-0.4**	**0.2**	*******	**-0.8**	**0.1**	******	**-0.4**	**0.2**
nup43	2E-08	***	-0.9	0.2	***	0.5	0.3	n.s.	-0.0	0.0
kpna2	1E-07	***	-1.1	0.1	n.s.	-0.2	0.1	n.s.	-0.2	0.7
nup85	**2E-07**	*******	**0.8**	**0.2**	*******	**0.6**	**0.4**	**n.s.**	**0.2**	**0.0**
nup54	**5E-07**	*******	**-0.8**	**0.1**	*****	**-0.3**	**0.2**			
kpna5	7E-07	***	-0.8	0.1	n.s.	-0.1	0.1	n.s.	0.1	0.1
ranbp1	**1E-06**	*******	**0.9**	**0.1**	*****	**0.3**	**0.0**	**n.s.**	**-0.3**	**0.3**
ranbp5	5E-06	***	0.8	0.4	n.s.	-0.1	0.2	n.s.	-0.0	0.1
pom121	6E-06	***	-0.6	0.1	*	-0.3	0.1	*	0.3	0.0
ranbp13	9E-06	***	-0.7	0.1	n.s.	-0.1	0.1			
xpo1	**2E-05**	*******	**0.6**	**0.3**	******	**0.5**	**0.2**	******	**0.4**	**0.4**
aladin	4E-05	***	0.9	0.0	n.s.	0.1	0.1	n.s.	0.1	0.1
ran	0.0003	***	-0.6	0.2	*	0.3	0.2	n.s.	-0.2	0.1
tpr	**0.0006**	*******	**-0.5**	**0.1**	*****	**-0.3**	**0.1**	**n.s.**	**0.2**	**0.2**
nup214	0.001	***	0.6	0.2	n.s.	0.1	0.2			
kpna7	0.002	***	-0.6	0.1	n.s.	0.1	0.1	n.s.	0.0	0.1
ranbp17	0.003	***	-0.5	0.1	n.s.	0.3	0.2	n.s.	0.1	0.1
cg1	0.007	***	-0.8	0.2	n.s.	0.1	0.2	n.s.	-0.2	0.5
nup107	0.007	***	-0.6	0.3	n.s.	0.1	0.2	n.s.	-0.1	0.2
tnpo3	0.007	**	0.4	0.1	*	-0.3	0.1			
nup62	0.02	**	0.4	0.2	n.s.	-0.2	0.1	n.s.	-0.2	0.1
rae1	0.02	***	-0.5	0.01	n.s.	-0.2	0.1	n.s.	-0.2	0.1
nupl1	0.02	***	-0.5	0.1	n.s.	-0.1	0.1	n.s.	0.1	0.1
ranbp6	0.05	**	0.4	0.5	n.s.	-0.3	0.1			
nup210	0.06	***	0.4	0.1	n.s.	0.1	0.2	n.s.	-0.2	0.1
nup37	0.06	***	0.5	0.2	n.s.	0.1	0.1	n.s.	-0.0	0.1
ranbp7	0.53	n.s.	0.3	0.6	n.s.	-0.3	0.1	n.s.	0.1	0.1
kpna3	0.55	*	-0.3	0.1	*	-0.3	0.0	n.s.	-0.1	0.1
ranbp4	0.58	*	-0.3	0.2	n.s.	-0.2	0.1			
ranbp8	0.73	**	0.4	0.1	n.s.	0.2	0.1	n.s.	-0.0	0.0
ndc1	0.77	*	0.4	0.2	n.s.	0.2	0.2	n.s.	0.1	0.1
xpo4	2.75	*	-0.3	0.1	n.s.	-0.1	0.3			
kpna6	5.82	n.s.	-0.2	0.1	n.s.	-0.2	0.3	n.s.	-0.1	0.2
nup188	7.18	n.s.	0.2	0.2	n.s.	-0.1	0.1	n.s.	-0.1	0.1
nup50	8.77	n.s.	-0.2	0.1	n.s.	-0.2	0.4	n.s.	0.0	0.1
nup35	9.29	n.s.	-0.2	0.2	n.s.	-0.1	0.1	n.s.	-0.0	0.1
kpna4	12.85	n.s.	-0.2	0.1	n.s.	-0.1	0.2	n.s.	0.0	0.3
rangrf	18.31	n.s.	-0.2	0.3	n.s.	-0.0	0.1	n.s.	0.0	0.1
xpo6	19.58	n.s.	0.1	0.0	n.s.	0.1	0.2	n.s.	0.1	0.3
nup93	25.74	n.s.	-0.1	0.1	n.s.	-0.1	0.1	n.s.	-0.0	0.1
ranbp9	27.67	n.s.	0.2	0.1	n.s.	0.1	0.1	n.s.	0.0	0.1
kpna1			-0.2	0.0						
nup88			-0.6	0.1						
ranbp3			0.4	0.1						
tnpo2			-0.9	0.3						
xpo5			0.6	0.1						
xpot			0.1	0.4						
