# Supplementary material for: The non-classical nuclear import carrier Transportin 1 modulates circadian rhythms through its effect on PER1 nuclear localization
Source: PLoS Genet. 2018 Jan 29;14(1):e1007189. doi: 10.1371/journal.pgen.1007189 (PMC5805371; doi:10.1371/journal.pgen.1007189)
Supplement: S2 Table — Clock protein-derived sequences containing at least one evolutionary conserved PY motif. (PDF) [file pgen.1007189.s011.pdf]

hnRNP A1 (268-305)	GNYNNQNQSSNFGPMKGGNFGGRSSG**PY**GGGGQYFAKPRNQGGYGGS
BMAL1 (97-146)	SLVPTCNAMSRKLDKLTVLRMAVQHMKTLRGATN**PY**TEANYKPTFLSDDEL
BMAL1 (533-581)	SSPGGKKILNGGTPDIPSSGLLSGQAQENPGY**PY**SDSSSILGENPHIGID
CRY1 (227-269)	ERKAWVANFERPRMNANSLLASPTGLS**PY**LRFGCLSCRLFYFKL
CRY2 (246-289)	RKAWVANYERPRMNANSLLASPTGLS**PY**LRFGCLSCRLFYYRLW
PER1 (294-337)	EKSVFCRIRGGPDRDPGPRYQPFRLT**PY**VTKIRVSDGAPAQPCC
PER1 (857-900)	SHPSPVPPSTPWPTPPATTPFPAVVQ**PY**PLPVFSPRGGPQPLPP
PER1 (909-952)	AFPAPLVTPMVALVLPNYLFPTPSSY**PY**GALQTPAEGPPTPASH
PER2 (219-262)	RDAFSDAKFVEFLAPHDVGVFHSFTS**PY**KLPLWSMCSGADSFT
PER2 (266-308)	EKSFFCRVSVRKSHENEIRYHPFRMT**PY**LVKVRDQQGAESQLCC
PER2 (1196-1239)	TGGLPAAIDVAECVYCENKEKGNICI**PY**EEDIPSLGLSEVSDTK
